# Machine learning identifies interacting genetic variants contributing to breast cancer risk: A case study in Finnish cases and controls

**DOI:** 10.1038/s41598-018-31573-5

**Published:** 2018-09-03

**Authors:** Hamid Behravan, Jaana M. Hartikainen, Maria Tengström, Katri Pylkäs, Robert Winqvist, Veli–Matti Kosma, Arto Mannermaa

**Affiliations:** 1 Institute of Clinical Medicine, Pathology and Forensic Medicine, and Translational Cancer Research Area, University of Eastern Finland, P.O. Box 1627, FI-70211 Kuopio, Finland; 2 Institute of Clinical Medicine, Oncology, University of Eastern Finland, P.O. Box 1627, FI-70211 Kuopio, Finland; 3 Cancer Center, Kuopio University Hospital, Kuopio, P.O. Box 100, FI-70029 Kuopio, Finland; 4 Laboratory of Cancer Genetics and Tumor Biology, Cancer and Translational Medicine Research Unit and Biocenter Oulu, Northern Finland Laboratory Centre Nordlab Oulu, University and University Hospital of Oulu, Oulu, Finland; 5 Biobank of Eastern Finland and Central Administration, Kuopio University Hospital, Kuopio, Finland

## Abstract

We propose an effective machine learning approach to identify group of interacting single nucleotide polymorphisms (SNPs), which contribute most to the breast cancer (BC) risk by assuming dependencies among BCAC iCOGS SNPs. We adopt a gradient tree boosting method followed by an adaptive iterative SNP search to capture complex non-linear SNP-SNP interactions and consequently, obtain group of interacting SNPs with high BC risk-predictive potential. We also propose a support vector machine formed by the identified SNPs to classify BC cases and controls. Our approach achieves mean average precision (mAP) of 72.66, 67.24 and 69.25 in discriminating BC cases and controls in KBCP, OBCS and merged KBCP-OBCS sample sets, respectively. These results are better than the mAP of 70.08, 63.61 and 66.41 obtained by using a polygenic risk score model derived from 51 known BC-associated SNPs, respectively, in KBCP, OBCS and merged KBCP-OBCS sample sets. BC subtype analysis further reveals that the 200 identified KBCP SNPs from the proposed method performs favorably in classifying estrogen receptor positive (ER+) and negative (ER−) BC cases both in KBCP and OBCS data. Further, a biological analysis of the identified SNPs reveals genes related to important BC-related mechanisms, estrogen metabolism and apoptosis.

## Introduction

Breast cancer is the second leading cause of cancer death in women with nearly 1.7 million new cases diagnosed in 2014. In Finland, BC accounted for 30.6% of all cancers in women resulting in 815 deaths out of 5008 BC patients (the Finnish Cancer Registry). The rapid growth in diversity and volume of genotyped genome-wide data collected from BC patients is opening unprecedented opportunities to adopt machine learning predictive modeling to identify risk factors, predict patient risk, and assist developing effective treatments to improve personalized clinical decision-making. Measuring an individual’s susceptibility to BC (or other complex diseases) prior to the diagnosis may determine who will eventually come down with the disease from those who will not. Identifying the BC-associated SNPs that reliably distinguish disease cases from healthy controls may be particularly useful in improving BC risk prediction^1^ and developing individual treatment strategies^2^.

Genome-wide association studies (GWAS) have successfully identified genetic variants with significant association with complex diseases spanning from BC^3^ to Alzheimer’s disease^4^. In GWAS, the idea is to identify genomic variants (SNPs) on the DNA, which explains the genetic component of the observed phenotype in genotyped people. In a typical GWAS study, we have in order of 10^5^–10^7^ SNPs and 10^2^–10^4^ samples, which indicate high dimensional features with possible correlation and a low sample size problem. Conventionally, standard hypothesis testing methods are adopted to measure the association between a single SNP with a disease by assigning the difference between frequencies of the alleles/genotypes between cases and controls, and measuring a *p*-value for each measured SNP individually. The *p*-values are then adjusted for multiple testing using, for example, the Bonferroni^5^ or Benjamini-Hochberg correction^6^ tests, and then the SNPs with *p*-values smaller than a pre-defined threshold are marked to have a high association^7^. This has several limitations as single SNPs have small effect size on observed phenotypes, while the explanatory power can be increased by the joint effect of (phenotype-associated) SNPs^8^. It also ignores the possible correlation/interaction among SNPs by analyzing one SNP at a time^8^.

Joint modeling of SNPs is challenging due to high dimensionality and small sample size. To date, population-based GWAS studies often use polygenic risk scoring (PRS)^9^ in which the disease risk for an individual is defined as the sum of the number of risk alleles across *m* disease-associated SNPs weighted by the effect size of each variant in forms of ${{\rm{PRS}}}_{i}={\beta }_{1}{g}_{1i}+\cdots +{\beta }_{m}{g}_{mi}$, where *g*_*si*_ is the number of effect alleles (0, 1 or 2) of SNP *s* for individual *i*, and *β*_*s*_ denotes the per-allele risk effect (odds ratio [OR] or hazard ratio (HR)) associated with the risk allele of SNP *s*. PRS assumes that the selected disease-associated SNPs are independent of each other and the risk effects are linear and additive^9^.

A significant body of works has recently devoted to penalized regression approaches to capture joint effects of SNPs^10–12^. These methods model a phenotype as a linear weighted sum of the genetic variants by applying a regularization penalty to constraint the magnitude of regression coefficients. This leads to a sparse set of SNPs that are predictive of the disease. The two most widely used penalized regression methods are lasso (least absolute shrinkage and selection operator)^13^ and ridge regression^14^. Both methods constraint the estimates of the regression coefficients towards zero relative to the maximum likelihood estimates. The lasso constraints sum of the absolute values of regression coefficients to be less than a fixed value (L1 penalty) encouraging sparse solutions. The ridge regression constraints sum of the squared regression coefficients (L2 penalty) resulting in small but non-zero regression coefficients.

Several studies have adopted penalized regression methods for GWAS assuming that each phenotype is an additive combination of latent SNPs. The performance of lasso was evaluated in a case-control study of coeliac disease with a large number of SNPs^15^. A ridge regression was used for differentiating causative from non-causative SNPs in linkage disequilibrium (LD)^16^. Other relevant studies are lasso for screening^12^, ridge regression for heritability estimation^11^ or lasso under stability selection for genotype-phenotype association study^10^. Although these approaches help to reduce overfitting and identify a number of disease-associated SNPs, they only capture linear dependencies between SNPs, and between SNPs and traits, and cannot capture non-linear SNP dependencies. As outlined by Moore *et al*.^17^, one of the significant challenges that must be overcome to successfully identify disease-associated SNPs in GWAS is the ability to model complex interactions, such as high-order non-linear interactions, between SNPs and disease susceptibility.

Among the multitude of choices for non-linear feature selection algorithms, extreme gradient tree boosting approach (XGBoost)^18^ has proven successful in several fields^19,20^, particularly in achieving state-of-the-art results in many Kaggle (https://www.kaggle.com/) machine learning challenges. XGBoost is rooted in the gradient boosted decision trees, which in contrast to lasso and ridge regression methods, incorporates complex non-linear feature interactions into prediction models in a non-additive form^18^. For example, in cancer research, integrating stochastic gradient boosting and cancer hallmark concepts has been found useful in determining cancer types based on copy number variants in the tumor founding clone^21^.

In this study, we propose a novel machine learning approach to identify group of interacting SNPs, which contribute most to the BC risk. Our proposed method is realized with an XGBoost model followed by an adaptive iterative SNP selection to capture multiple-way SNP-SNP interactions and identify group of interacting SNPs, which achieve high BC risk prediction accuracy. In contrast to PRS, the proposed method incorporates complex non-linear SNP interactions into the BC risk prediction model in a non-additive form assuming dependencies among the SNPs and between the SNPs and the trait. The resulting method is simple yet very effective to capture the optimal ways of combining candidate BC risk-predictive SNPs to achieve high BC risk prediction accuracy for different populations (here, Kuopio and Oulu).

We have demonstrated our approach on the Kuopio Breast Cancer Project (KBCP)^22^ and the Oulu Breast Cancer Study (OBCS) (University of Oulu/Oulu University Hospital, 2004)^23^ sample sets. We compared the proposed approach with a system trained on 51 known BC-associated SNPs^24,25^, a PRS-derived model and a number of conventional machine learning methods already used in GWAS to identify disease-associated SNPs. We then investigated the predictive potential of the identified SNPs in classifying ER status. Finally, we carried out a gene interaction analysis to gain biological insight into the identified SNPs.

## Proposed Approach

**Figure 1:**
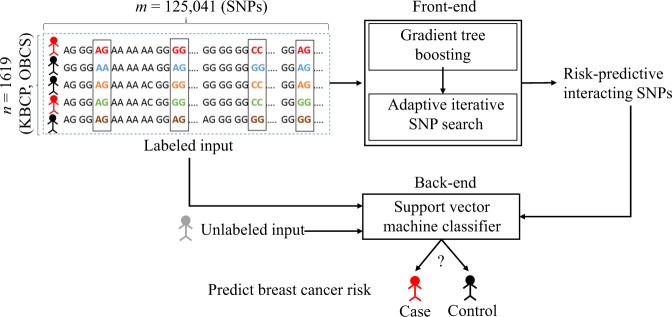
An overall representation of the proposed BC risk prediction approach using identified risk-predictive interacting SNPs. We propose an effective machine learning approach to identify group of interacting SNPs, which contribute most to the BC risk. The identified SNPs are then used to predict the BC risk for an unknown individual in the back-end.

Figure 1 illustrates a general overview of the SNP selection process for the BC risk prediction used in the present study. The front-end is a SNP selection process using an XGBoost model followed by an adaptive iterative SNP search to capture an optimal group of interacting SNPs with high BC risk-predictive potential. The SNPs identified in the front-end are then used to predict the BC risk using a support vector machine (SVM) classifier in the back-end. In the following, we describe the individual components of the proposed approach, in detail.

### Gradient tree boosting

Boosting is an effective ensemble learning algorithm in which weak classifiers are added sequentially to correct the errors made by existing classifiers towards building a strong classifier. XGBoost technique is a fast and an efficient implementation of the gradient tree boosting method described in detail in Supplementary file Section ‘Gradient tree boosting’, whose parameters are fully tunable. The implementation is available as a library at https://xgboost.readthedocs.io/. In this study, XGBoost is used to evaluate the importance of SNPs on a BC risk prediction task by providing an initial list of candidate BC risk-predictive SNPs. We call this process the first module of our proposed approach. We used the average of feature importances (a.k.a. “gain”) provided by the gradient tree boosting method (see Supplementary equation (S12)), as the contribution of each SNP to the BC risk. More details on the XGBoost hyperparameter tuning will be provided in the experimental section.

### An adaptive iterative SNP selection algorithm

Based on the XGBoost initial candidates of the BC risk-predictive SNPs (first module), the second module of our proposed approach uses the candidate SNPs for an adaptive iterative search (see Algorithm 1) to capture the optimal ways of combining candidate SNPs to achieve high BC risk prediction accuracy on a validation data. First, candidate SNPs are sorted in descending order based on their importance scores generated from an XGBoost model trained using the whole available SNPs. The SNPs with the highest importances are regarded as top SNP list and the SNPs with the lowest importances are regarded as bottom SNP list. After selecting the top and the bottom SNP lists, we then re-rank the two SNP lists using two XGBoost models independently trained on the SNPs of the top and the bottom SNP lists. We then substitute the highest-/lowest-ranked SNP from the bottom/top list with the lowest-/highest-ranked SNP from the top/bottom list and gradually increase the number of SNPs from these two lists before list overlap is observed. We call this process the second module of our proposed approach. This process of re-ranking has the effect of capturing a wide range of SNP-SNP interaction patterns, and consequently, identifies group of interacting SNPs, which contribute most to the BC risk, and places them on the top of the SNP list.

### Support vector machine

Support vector machine is a discriminative supervised classifier initially introduced by Cortes and Vapnik^26^. Given labeled training data, an SVM finds the maximum margin separation hyperplane (decision boundary) to classify training examples such that it generalizes well to the unseen data. In this study, SVM was trained to distinguish the BC cases (positive samples) and healthy controls (negative samples) using the *S* top-ranked SNPs as feature vectors and a linear kernel defined as^26^:

1 $$\kappa ({{\rm{x}}}_{i},{{\rm{x}}}_{j})={{\rm{x}}}_{i}^{\top }{{\rm{x}}}_{j},$$

where, x_*i*_ and x_*j*_ are two SNP feature vectors, and $\top$ denotes the transpose operation.

**Algorithm 1:**
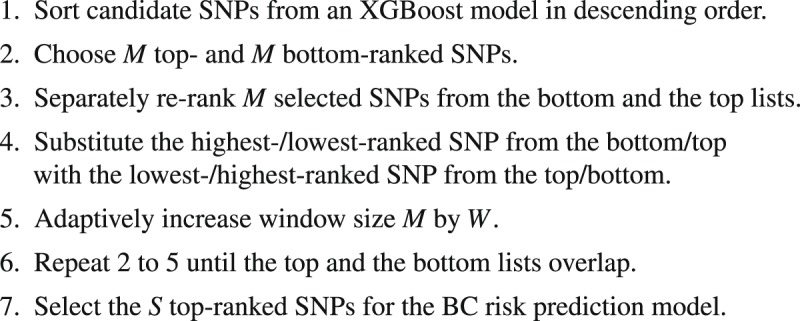
An adaptive iterative SNP selection process to capture a wide range of SNP-SNP interaction patterns.

## Experimental Set-Ups

### Sample sets

To perform the BC risk prediction task, we used the KBCP and the OBCS sample sets. Genotyping was done using a custom Illumina array iCOGS with 211,155 SNPs. Genotyping, allele calling, and quality control for the Breast Cancer Association Consortium and iCOGS study are described in detail in Michailidou *et al*.^25^. Patient samples were obtained with informed written consent. The KBCP sample set including all methods have been approved by the ethical committee of the University of Eastern Finland and Kuopio University Hospital. The OBCS sample set including all methods have been approved by the Finnish Ministry of Social Affairs and Health, and the ethical committee of Oulu University Hospital.

The KBCP controls were carefully selected from healthy individuals of the Savo region in Eastern Finland matching individually to each BC case by age and long-term place of living, thus originating from the same genetic background as the KBCP cases. The OBCS controls were collected from blood donors for the Finnish Red Cross without taking into consideration the demographic and the genetic background of donors.

Table 1 shows the distribution of the BC cases and controls as well as the ER+ and ER− subtypes in the KBCP and the OBCS sample sets used in this study. We excluded missing genotype values from the SNP data. The final dataset consisted of 125,041 SNPs in both the KBCP and the OBCS sample sets. SNPs are encoded using an additive encoding scheme^27^. The additive encoding represents each SNP through the minor allele count in which homozygous major, heterozygous and homozygous minor are encoded as 0, 1, and 2, respectively.

**Table 1:** Distribution of the BC cases and controls, the ER+ and ER− subtypes in the KBCP and the OBCS sample sets.

Sample sets	#Cases	#Controls	#Individuals	#ER+	#ER−
KBCP	445	251	696	316	101
OBCS	508	415	923	407	100

### Evaluation strategy

**Figure 2:**
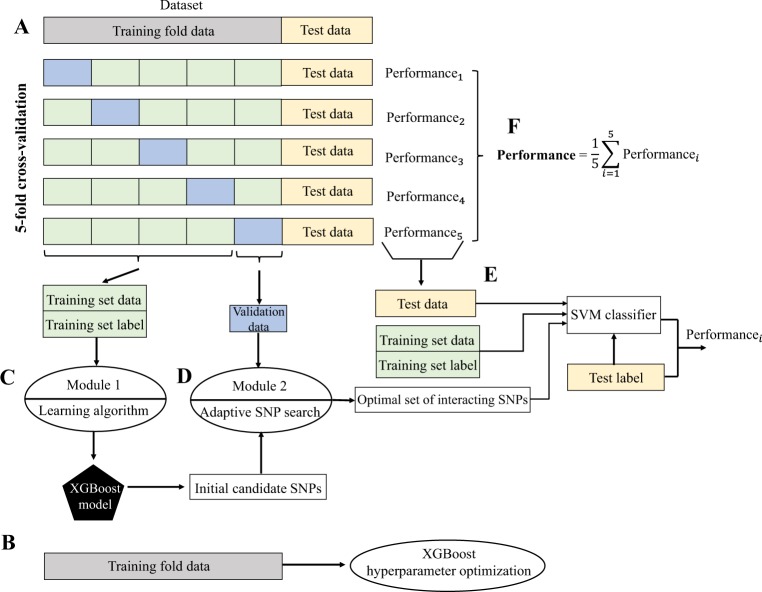
Visual representation of the proposed SNP selection approach in a BC risk prediction task. (**A**) Partitioning the genotyped data into training fold and test data with 4:1 proportion. The training fold data is further partitioned using a 5-fold stratified CV: one fold (validation data) is used for evaluating the set of identified SNPs produced by the module 2 and the remaining 4 folds are merged into a training set data for XGBoost model training and finding initial candidate BC risk-predictive SNPs (module 1). (**B**) Using training fold data for XGBoost hyperparameter optimization. (**C**) Module 1: using training set data to learn an XGBoost model and produce initial list of candidate BC risk-predictive SNPs. (**D**) Module 2: An adaptive iterative SNP selection process using the initial list of candidate SNPs obtained from **C** and the validation data. In this process, SNPs are re-ranked (see Algorithm 1) and the top interacting SNPs yielding the best BC risk prediction accuracy on the validation data are selected. (**E**) The top identified interacting SNPs from (**D**) are adopted to predict the BC risk on the test data using an SVM classifier. (**F**) Performances are averaged to obtain the final BC risk prediction accuracy across the test data. Same individuals are not used in the training, validation and test sets.

To overcome the lack of suboptimal amount of genotyped BC data to train high-performance BC risk prediction model, we have evaluated our proposed method in 10 repetitions of 5-fold cross-validation (CV). We used the KBCP genotyped data to optimize the XGBoost hyperparameters. Figure 2 shows a visual overview of our proposed SNP selection process and the BC risk prediction task. At each repetition round, the genotyped data is randomly split into non-overlapping training fold and test data with 4:1 ratio, keeping class frequencies balanced. The test data is used only to evaluate the final prediction accuracy and is not used in the SNP selection process. The training fold data is used to optimize the XGBoost hyperparameters. The training fold data is further partitioned into 5 folds using stratified CV: one part (validation data) is used for evaluating the group of interacting SNPs identified from the second module of the proposed approach and the remaining 4 parts are merged into a training set data for XGBoost model training and finding initial candidates of BC risk-predictive SNPs (first module). The identified group of interacting SNPs are then used to predict the BC risk on the test data. Individual accuracies are finally averaged across all iterations to get the final prediction accuracy.

It is worth noting that following the above iterative process, various groups of interacting SNPs will be computed (10 (iterations) × 5 (cv) = 50 groups). SNPs may overlap among groups. This iterative partitioning allows placing BC patients in various training, validation, and test folds accounting for possible heterogeneity among BC cases and consequently, identifying corresponding BC risk-predictive SNPs for each partition.

We chose to optimize the following XGBoost hyperparameters (i) number of decision trees — the boosted trees are constructed sequentially by adding new trees (weak learners) to the model while each new tree attempts to correct the errors made by the previous sequence of trees. The model often reaches a point, where the addition of new trees does not improve the model performance, (ii) size of decision trees (tree depth) — is used to control over-fitting as trees with higher depth generally learn too many details from the training samples, (iii) learning rate (shrinkage factor) — slows down the learning in the gradient tree boosting model by reducing the impact of each individual tree in the estimates and leaving space for future trees to improve the model, and finally, (iv) subsampling rate — the fraction of samples to be selected from the training data to create each tree. The selection is performed by random sampling without replacement. This simple technique (a.k.a. “stability selection”) adds variance to the ensembled estimation by allowing slightly different trees to be constructed from the random subset of the training data.

A grid search over the triple of the number of decision trees, the size of decision trees and the learning rate is first performed within each iteration using the training fold data, then, the subsampling rate is optimized following the previously found optimal hyperparameters.

### Evaluation metrics

We use the precision-recall curve and AP, widely-used evaluation metrics, to compare the performances of the different methods in discriminating the BC cases and controls on the test data. The precision-recall curve illustrates the trade-off between precision and recall at different cut-off points^28^. Precision and recall are defined as^28^:

2 $${\rm{Precision}}=\frac{TP}{TP+FP}$$

3 $${\rm{Recall}}=\frac{TP}{TP+FN},$$

where, TP = number of true positives, TN = number of true negatives, FP = number of false positives and FN = number of false negatives.

Average precision is a single number summarizing the precision-recall curve by computing the weighted mean of the precisions achieved at each cut-off points, using the increase in recall from the previous cut-off point as the weight^29^:

4 $${\rm{AP}}=\sum _{i}\,({{\rm{recall}}}_{i}-{{\rm{recall}}}_{i-1})\times {{\rm{precision}}}_{i}$$

where, recall_*i*_ and precision_*i*_ are the precision and recall at the *i*-th threshold. Average precision denotes the average area under the precision-recall curve between 0 (worst) and 1 (best)^28^. Mean average precision evaluates the prediction model performance by averaging AP across multiple test subsets.

### Baseline models for performance comparison

For comparison, we derived PRSs from 51 previously reported BC-associated SNPs^24^ and their published iCOGS OR^25^. From the 92 published BC-associated SNPs in^24^, only 51 SNPs existed in our SNP discovery set in both KBCP and OBCS sample sets. Recently, Michailidou *et al*.^30^ has published more than 100 BC-risk associated SNPs, which we will consider in our next study. A list of SNPs and ORs used in the PRS models can be found in Supplementary Table S1. To evaluate the ability of the PRS to discriminate between the BC cases and controls, we computed the recall and precision at every possible PRS cut-off points. We then estimated the AP from the precision-recall curve, integrating over all the possible cut-off points. We also treated the 51 BC-associated SNPs as feature vectors and fed them into the SVM classifier for the BC risk prediction. This system is denoted as ‘Literature SNPs’ in the result section.

Additionally, we compared our proposed SNP selection approach with three classical feature selection methods, i.e. L1, L2 and elastic net (L1 ratio = 0.4) regularized logistic regressions, with arbitrary inverse of regularization strength *C* = 0.7, following the same data partitioning and the back-end illustrated in Fig. 2.

### Implementation details

We implemented the proposed approach with XGBoost 0.6a2 and Python Scikit-Learn 0.18.2 using a Linux machine equipped with 42 CPUs and 400 GB memory. The implementation source codes are freely available at https://github.com/hambeh/breast-cancer-risk-prediction.

For the variant analysis, we used Ensembl release 91^31^ to characterize the variants. Overlapping genes were identified within 5,000 bp upstream and downstream of each variant. To search for biological evidence of the important combination and interactions of SNPs identified in this study, we created a network using a list of genes associated with the variants and esyN^32^ (www.esyN.org). esyN is an open source bioinformatics web-tool for visualizing interaction data, in which nodes represent biological entities (e.g. gene, protein, molecule) and the interactions between them are represented by edges connecting the nodes. esyN is primarily written in the javascript language, using the following libraries: cytoscape.js^33^, intermine^34^, jQuery^35^, angularJS^36^, underscore.js^37^.

## Results and Discussion

### Optimizing XGBoost hyperparameters

We first optimized the XGBoost hyperparameters in the context of BC risk prediction task. For this purpose, we used the negative log-loss of the model accuracy computed by Supplementary equation (S3) and the KBCP genotyped data following the procedure illustrated in Fig. 2. The results are summarized in Supplementary Table S2 and detailed in Supplementary Fig. S1 for each iteration. As expected, fewer boosted trees are required with the increase in the tree depth. Deeper individual trees resulted in overfitting of the training data, which would be aggravated with more boosted trees as outlined by Friedman^38^. The optimal tree depth was found to be 2 for all except the fifth iteration, although there was practically little difference between using tree depth = 2 or tree depth = 4 for this iteration. We also found that increasing the learning rate degrades the model accuracy. The optimal value of learning rate was found to be 0.01 for all iterations. Regarding the stochastic gradient boosting, the best results are achieved with the aggressive subsampling of the training data, such as 40% to 60%, which is in line with the findings by Friedman^38^.

### Breast cancer risk prediction via adaptive iterative search

**Figure 3:**
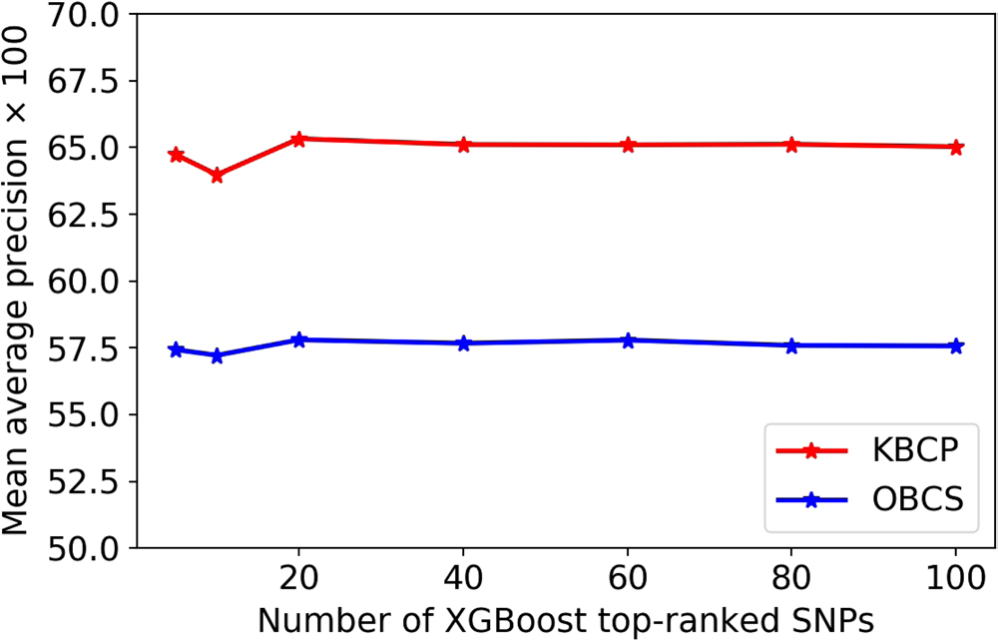
BC risk prediction as a function of number of XGBoost top-ranked SNPs. No improvement is observed by increasing the number of XGBoost top-ranked SNPs.

Figure 3 displays the BC risk prediction accuracy in terms of mAP, as a function of XGBoost top-ranked SNPs on the KBCP and the OBCS validation data, respectively. Even if no considerable change is observed by changing the number of XGBoost top-ranked SNPs, each individual selected SNP contributes to the BC risk prediction model. It is the second module of the proposed approach, i.e. the adaptive iterative SNP search, which captures the optimal interacting SNPs (from the XGBoost provided list of important SNPs), that contribute most to the BC risk prediction.

**Figure 4:**
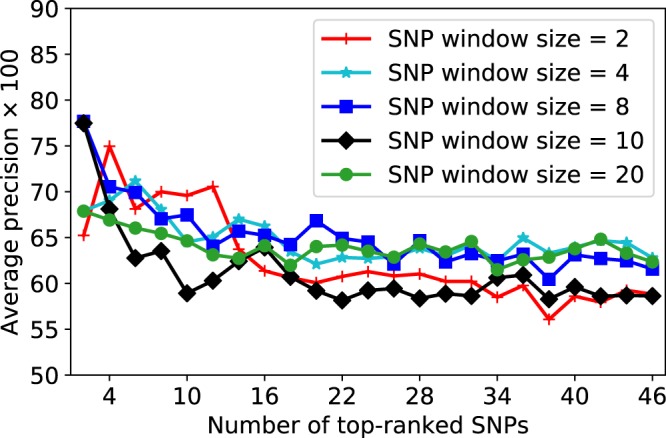
BC risk prediction as a function of the number of the top-ranked SNPs for arbitrary SNP window sizes on a validation subset. SNPs are sorted based on their BC risk-predictive importance score from the highest to the lowest on the x-axis. The adaptive iterative search algorithm with arbitrary SNP window sizes tend to group and sort the SNPs with the highest BC risk-predictive potential.

Next, Fig. 4 shows the BC risk prediction accuracy on a validation data via the increase of the number of top-ranked SNPs for arbitrary SNP window sizes. We can see that the proposed adaptive iterative SNP search algorithm, which is intended to capture the optimal SNP-SNP interaction patterns, tends to group and sort the SNPs with the highest BC risk-predictive potential. For example, the top 4 SNPs together yielded the best BC risk prediction accuracy with AP of 74.96 for window size = 2. Similarly, the first-ranked SNP resulted in the highest prediction accuracy with AP of 79.00 for window size = 8.

We have similarly applied this adaptive search over the initial candidate BC risk-predictive SNPs provided by the XGBoost model (see module 1 in Fig. 2) and found the corresponding top-ranked SNPs for each validation data. In fact, the final group of interacting top-ranked SNPs for each validation data is determined by evaluating several window sizes (*M* = 2, 4, 6, 8, 20, 30) with adaptive window size increases (*W* = 1, 2, 3, 4, 5). A summary of the obtained values for the optimal window sizes and adaptive window size increases is given in Supplementary Table S3 for each round of data partitioning. We can see that smaller SNP window sizes are often marked as optimal values. As an example, SNP window size = 2 is found optimal in 17 out of 50 splits. Similarly, the adaptive window size increase = 1 is found optimal in 15 out of 50 splits.

### Breast cancer risk prediction in the KBCP and the OBCS sample sets

**Figure 5:**
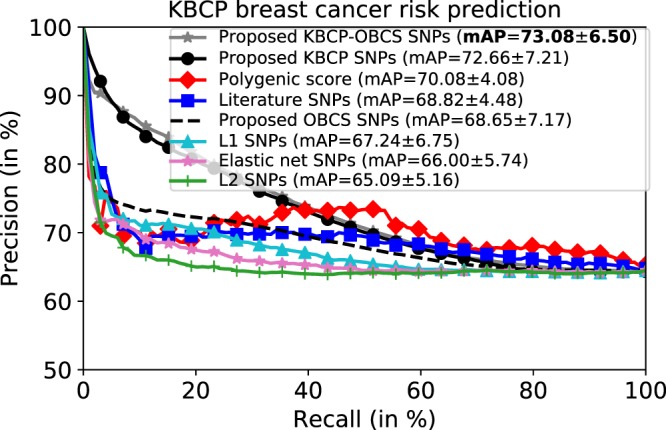
Average precision-recall curve of the KBCP BC risk prediction task. Each plot corresponds to the prediction results obtained by the SNPs identified from one of the methods. The prediction accuracy of the proposed method on the KBCP data is separately reported for the SNPs identified from the KBCP, the OBCS and the merged KBCP-OBCS sample sets. The penalized logistic regression methods are trained using the KBCP sample set. Using the identified SNPs from the merged KBCP-OBCS genotyped data, the proposed method achieves the best BC risk prediction results compared to the baseline systems. The number after ± denotes standard deviation. High standard deviations are due to multiple subset selections.

Figure 5 illustrates the precision-recall curve comparison between the proposed SNP selection approach and the five baseline methods on the KBCP test data when the models are trained from the KBCP data. The results indicate that using the KBCP identified SNPs, the proposed SNP selection approach outperforms the baselines in discriminating the KBCP cases and controls in terms of mAP. The proposed approach achieves mAP of 72.66 in discriminating the BC cases and controls on the KBCP test data. From the baselines, the system based on the PRS obtains the highest performance with mAP of 70.08. From the penalized logistic regression methods, the system based on L1 penalty attains the highest prediction accuracy with mAP of 67.24.

To measure the contribution of the adaptive iterative SNP search to capture the optimal group of interacting SNPs, we excluded the adaptive iterative search and used all the candidate KBCP SNPs produced by the XGBoost model to perform the BC risk prediction on the KBCP test data. The prediction accuracy degrades to 65.04, that is a 10% relative reduction in mAP, highlighting the importance of capturing the optimal SNP-SNP interactions by the adaptive iterative SNP search in discriminating the KBCP cases and controls.

It is instructive to recall the order of computations: XGBoost model training → Obtaining initial candidates of BC risk-predictive SNPs → Performing adaptive iterative search over the candidate SNPs → Capturing group of interacting SNPs with the highest BC risk-predictive potential → Predicting BC risk using the identified interacting SNPs and an SVM classifier.

**Figure 6:**
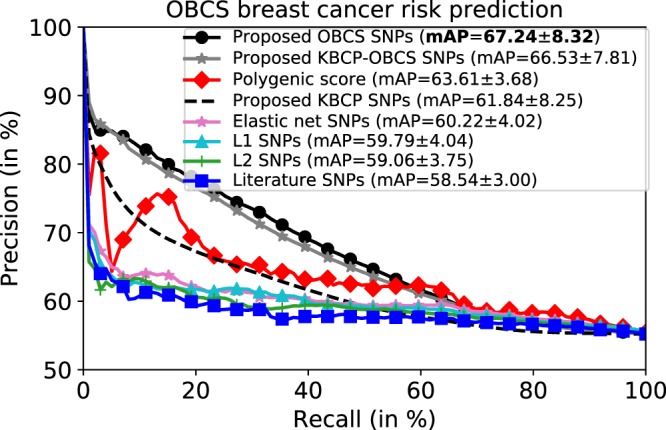
Average precision-recall curve of the OBCS BC risk prediction task. The prediction accuracy of the proposed method on the OBCS data is separately reported for the SNPs identified from the KBCP, the OBCS and the merged KBCP-OBCS sample sets. The penalized logistic regression methods are trained using the OBCS data. The proposed method attains the best BC risk prediction results, when the SNPs are identified from the OBCS data.

Up to this point, we have focused on the KBCP data to optimize the XGBoost model hyperparameters and find the optimal group of interacting SNPs, which best discriminate the KBCP cases and controls. We now use the optimal hyperparameter values and the SNPs identified from the KBCP data to predict the OBCS cases and controls as a validation study in 10 repetitions of 5-fold CV. The results are illustrated in Fig. 6. Using the KBCP identified SNPs, the proposed method marginally outperforms the systems based on the penalized regression methods (SNPs identified from the OBCS data for the penalized regression systems) and the literature SNPs, however, it underperforms the PRS-derived model by 3% mAP relative reduction, which is understandable considering that the important SNPs were identified and hyperparameters were optimized both on the KBCP training fold data. Interestingly, by using the OBCS data to train the BC risk prediction model and obtain OBCS-specific BC risk-predictive SNPs, the mAP increases to 67.24, indicating 5% and 8% relative mAP improvements, respectively, over the PRS-derived model and the system, which uses the identified KBCP SNPs in discriminating the OBCS cases and controls. Similar to the KBCP task, excluding the adaptive iterative SNP search degrades the BC risk prediction accuracy to 58.28, that is a 13% relative reduction in mAP, which further highlights the contribution of the adaptive iterative SNP search in the proposed BC risk prediction task.

To evaluate robustness and overfitting of the proposed method, Table 2 summarizes the prediction performance on the training, validation and test sets for both KBCP and OBCS sample sets using the proposed KBCP and OBCS SNPs, respectively. The results show that the proposed method does not overfit the training data and performs favorably in both validation and test sets in the two datasets.

**Table 2:** BC risk prediction accuracy in terms of mAP in the training, validation and test sets for both KBCP and OBCS sample sets using the proposed KBCP and OBCS SNPs, respectively. The results indicate the robustness of the proposed method in discriminating BC cases and controls in both sample sets.

Dataset	Training set	Validation set	Test set
KBCP	76.78 ± 7.01	74.54 ± 6.57	72.66 ± 7.21
OBCS	73.96 ± 9.33	69.49 ± 7.21	67.24 ± 8.32

A principal component analysis (PCA)^39^ of the BC cases over all 125,041 available SNPs further indicates the population-specific variation between the KBCP (Kuopio population) and the OBCS (Oulu population) genotyped data (see Supplementary Fig. S2). As outlined by Kerminen *et al*.^40^, Finland represents a highly geographically clustered genetic structure with little overlap between the populations due to specific population history of the Western and the Eastern Finland.

We further selected the OBCS cases and controls that overlap with the KBCP samples in terms of genetic structure following the PCA plots shown in Supplementary Figs. S4 and S5 respectively for the OBCS cases and controls. This accounts for 142 OBCS cases and 87 controls, which are closely related to the KBCP cases and controls, respectively. Using the proposed identified KBCP SNPs, the BC risk prediction accuracy increases to 70.06 in terms of mAP in the OBCS samples overlapping with the KBCP genotyped data, which is in line with the 72.66 mAP achieved on the KBCP test data using the same set of SNPs (Fig. 5, second plot). This indicates that the reduced predictive power of the proposed identified KBCP SNPs when applied to OBCS data is likely due to the differences in the underlying genetic structure of these two populations.

We also investigated the effect of merging the KBCP and the OBCS sample sets into a single sample set to identify the BC risk-predictive SNPs denoted as KBCP-OBCS SNPs in Figs. 5 and 6. This resulted in identifying 136 interacting BC risk-predictive SNPs, which is considerably lower than 407 and 563 interacting SNPs identified from the KBCP and the OBCS sample sets, respectively. As shown in Supplementary Fig. S2, the merged KBCP and OBCS genotyped data shows together a more dense cloud structure in the PCA 2D projection space than individually. Furthermore, the subtypes of the cancer cases could be more homogeneous in the combined analysis than in the individual sample sets. These would indicate less variance among the cases in the merged sample set, which might result in the lower number of optimal interacting SNPs. Results in Figs. 5 and 6 further show that regardless of which genotyped data to be used, the proposed approach compares favorably with the PRS-derived model in discriminating the BC cases and controls in all cases except, when the identified KBCP SNPs are used in the OBCS BC risk prediction task and vice versa. When the two sample sets are merged, the proposed approach and the PRS-derived model obtain mAP of 69.25 and 66.41, respectively, in discriminating the BC cases and controls on the merged data.

### The predictive potential of the identified SNPs in classifying estrogen receptor status

**Figure 7:**
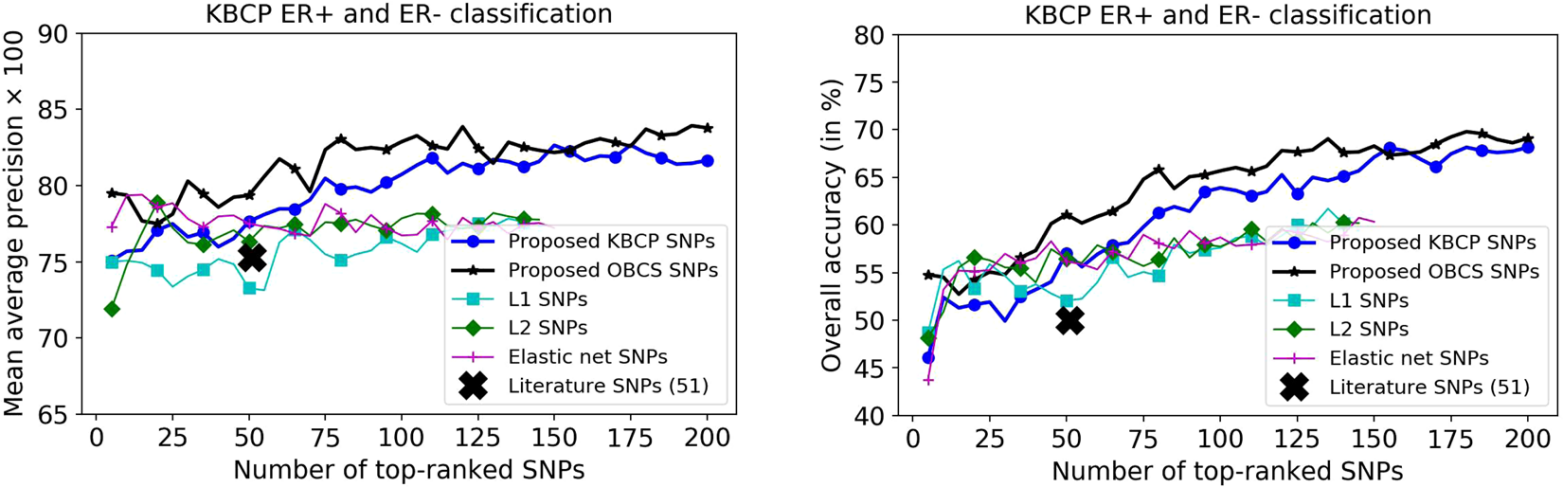
KBCP ER+ and ER− classification using the BC risk-predictive SNPs identified by the proposed and the baseline methods. SNPs are sorted on the x-axis based on their importance score from the highest to the lowest provided by an XGBoost model in discriminating ER+ and ER− BC cases for all the methods. XGBoost ranking discards SNPs, which do not contribute to the ER subtype classification. Increasing the number of top-ranked SNPs improves the ER+ and ER− classification accuracy. The improvement is more prominent for the SNPs identified from the proposed method. Overall accuracy denotes the percentage of correctly classified instances. L1, L2 and elastic net SNPs relate to the identified SNPs from the penalized logistic regression respectively with L1, L2 and elastic net penalties.

**Figure 8:**
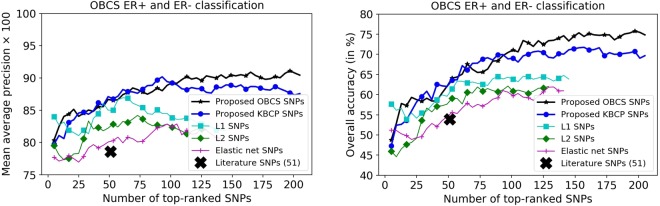
OBCS ER+ and ER− classification using the BC risk-predictive SNPs identified by the proposed and the baseline methods. Similar to the KBCP ER experiment, increasing the number of top-ranked SNPs improves the ER+ and ER− classification accuracy. The improvement is more prominent for the SNPs identified from the proposed method.

**Figure 9:**
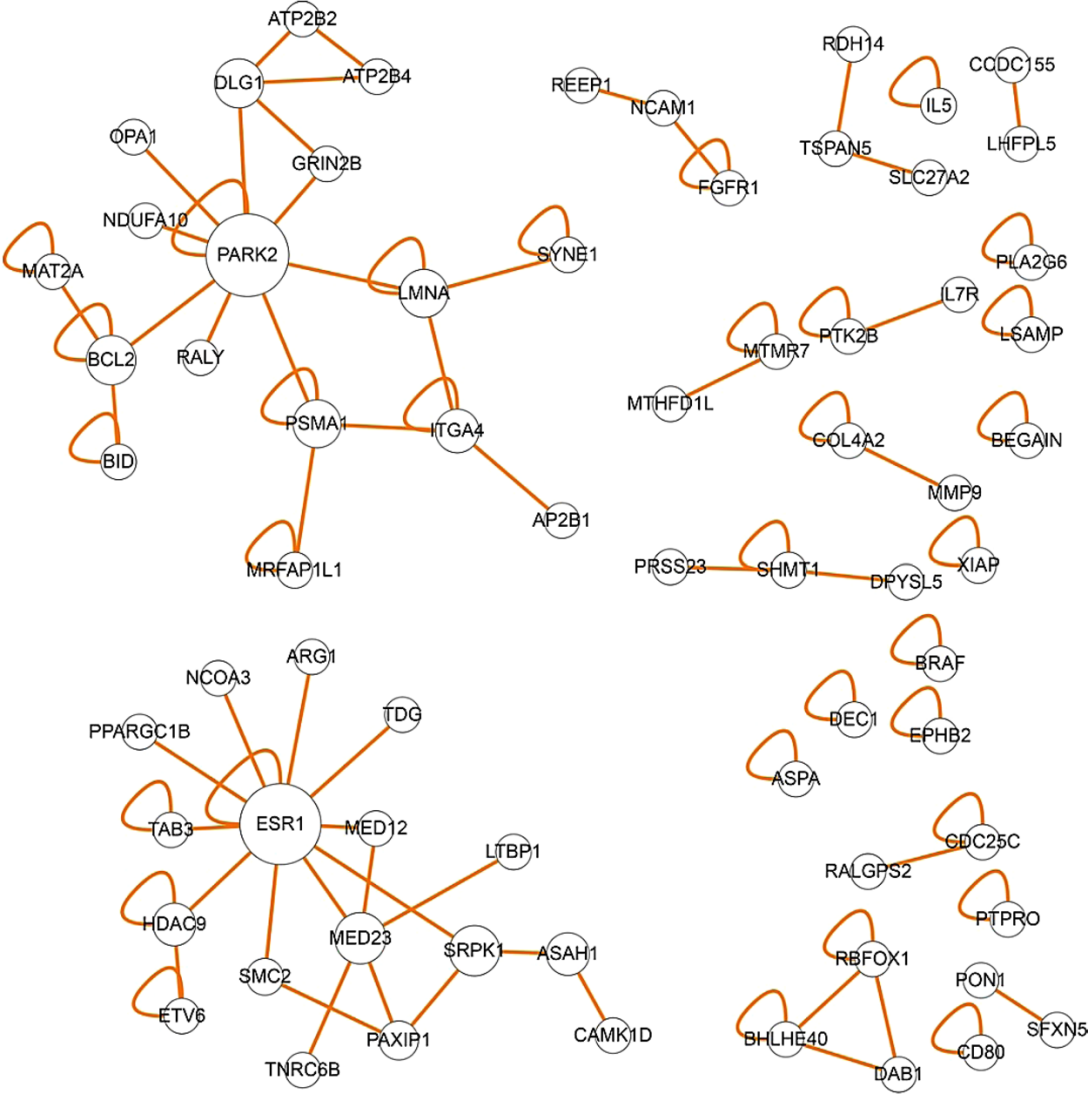
Gene interaction map of the identified KBCP SNPs reveals that the proposed approach can identify interacting genetic variants truly biologically relevant to the BC risk.

Breast cancer is a heterogeneous disease consisting of many subtypes of which the ER+ and ER− subtypes are the key ones^41^. Now, we turn our attention to evaluating the predictive power of the identified SNPs to classify ER+ and ER− status in the BC cases. For this purpose, we concatenated the identified BC risk-predictive SNPs individually for each method to classify ER+ and ER− status of the cases, using a 10-fold CV. Results are shown in Figs. 7 and 8, respectively for the KBCP and the OBCS data. As illustrated, increasing the number of top-ranked SNPs improves the ER+ and ER− classification accuracy both for the KBCP and the OBCS data. This improvement is prominent for the risk-predictive SNPs identified by the proposed method with the maximum mAP of 84.15 and 91.20 respectively, for the KBCP and the OBCS data, highlighting their predictive potential in discriminating ER+ and ER− cases compared to other baseline identified SNPs. From the 200 identified KBCP SNPs by the proposed method, 10 were found in the ER gene (ESR1) interaction network illustrated in Fig. 9.

### Analysis of the identified interacting SNPs and associated genes

Besides outperforming the classical SNP selection approaches as well as the PRS-derived model, the proposed technique provides a framework for tools to study biological insight of the identified SNPs. A total of 300, 587 and 118 genes could be associated with the identified 407 KBCP, 563 OBCS and 136 KBCP-OBCS SNPs, respectively. Details of the genes associated with the identified interacting SNPs from each of the sample sets as well as the literature SNPs can be found in Supplementary Tables S4–S7.

The total number of SNPs overlap between the identified KBCP and the OBCS SNPs is 3 (rs1520148, rs6570423 and rs9365352). Also, we found that for the SNPs locating on the same chromosomes, 141 out of 407 identified KBCP SNPs and 148 out of 563 identified OBCS SNPs are within 1 Mb chromosomal region respectively from the identified OBCS and KBCP SNPs, which may indicate possible association among the identified SNPs from these two sample sets. The gene interaction maps of the identified KBCP (Fig. 9) and OBCS (Supplementary Fig. S7) SNPs further show that the KBCP and OBCS SNPs point to a number of identical networks, such as PARK2 and ESR1. Note that the proposed approach is trained to find the group of interacting SNPs that contribute most to the BC risk rather than find the maximum number of shared SNPs between the two sample sets. Moreover, this experimental evidence has been drawn from the low-sample size datasets, which indeed can affect the frequency of rare alleles within each sample set as well as the BC risk prediction performance, and together with the sample set genotyped differences (see Supplementary Figs. S2 and S3 respectively for the PCA of the cases and healthy controls), might partially explain the variation observed between the identified interacting SNPs for the individual as well as the merged sample sets.

From the KBCP associated gene interaction map (Fig. 9), we also found several separate networks of which ESR1–linked and PARK2- and BCL2–linked networks were the most prominent. PARK2 has recently been linked with BCL-XL-dependent control of apoptosis^42^. As apoptosis and estrogen-related entities are constitutional factors in tumorigenesis, we believe this result shows evidence that our approach can identify truly biologically relevant group of interacting genetic variants. Note that the identified interacting SNPs might also link to the biological networks with no (known) effect on BC risk-related mechanisms.

A number of strategies of “predictive genomic” have been published to predict personalized drug targets, drug resistance, and metastasis for cancer patients, as well as cancer risks. Gao *et al*.^43^ used gene signature sets to successfully predict prognosis of stage II colorectal cancer patients. Li *et al*.^44^ developed an algorithm that identified prognostic markers focusing on metastasis-driving gene expression signals. Application of the algorithm to BC samples identified prognostic gene signature sets for both ER+ and ER− subtypes. Use of cancer hallmarks as framework in cancer biomarker identification have been reviewed by Wang *et al*.^45^. Strategies of using this framework in conjunction with genome, transcriptome and epigenome data to predict outcome of cancer patients, as well as cancer risks for healthy individuals could have substantial impact on diagnosis, personalized treatment and personalized prevention of cancer. Indeed, the proposed approach in this study, which is free from pre-selection of important cancer-related entities, can be integrated into hallmark-based strategies to further select true biologically-relevant interacting factors (e.g. SNPs) contributing to cancer risk.

The gene interaction network of 51 literature SNPs (Supplementary Fig. S6) shows that the literature SNPs form individual entities with no genetic interactions. In addition to the ESR1 and the PARK2 networks, a number of important cancer-related entities, such as EGFR and MAPK1, were also found prominent in the OBCS gene interaction map illustrated in Supplementary Fig. S7. EGFR pathway has an impact on metabolic pathways in cancer cells^46^. In BC, EGFR promotes breast tumor growth and metastasis^47^. Regarding MAPK1, Si *et al*.^48^ have recently shown that silencing of MAPK1 can impair the proliferation of BC cells and reduce their drug resistance. The gene interaction analysis of the identified KBCP-OBCS SNPs further points to HDAC3- and ESR1- linked networks (see Supplementary Fig. S8). HDAC3 is essential for maintaining genome stability and efficient DNA repair and replication^49^. Genomic instability is regarded as a key characteristic of most cancers.

Finally, while it is correct that the differences between the KBCP and the OBCS controls might also affect the identified interacting SNPs (see Supplementary Fig. S3 for PCA of the healthy controls), we still observe SNPs which truly relate to the important BC relevant pathways as shown in the gene interaction maps (Fig. 9, Figs. S7 and S8). Moreover, the identified interacting SNPs perform favorably against the widely-used additive and PRS-derived models to predict the BC risk. Incidentally, the developed method in this study could also be useful in detecting interacting SNPs (or other types of data, such as protein interactions and microRNAs) for other diseases.

## Conclusion

In this study, we have developed a simple yet effective machine learning based approach to identify group of interacting SNPs, which contribute most to the BC risk.

The leading idea is to take advantage of non-linear feature selection algorithms by assuming dependencies among the SNPs and between the SNPs and the BC risk. To this end, we adopted a gradient tree boosting method followed by an adaptive iterative SNP search to capture complex SNP-SNP interaction patterns and consequently, obtained group of interacting SNPs, which yielded high BC risk prediction accuracy within the SVM-based framework.

Experimental results on two BC cohorts, namely the KBCP and the OBCS, have demonstrated the effectiveness of the proposed approach, which compares favorably with the classical linear penalized logistic regression methods and a PRS model derived from the 51 known BC-associated SNPs, in a small sample set problem. The proposed approach achieves mAP of 72.66, 67.24 and 69.25 in discriminating BC cases and controls in the KBCP, the OBCS and the merged KBCP-OBCS sample sets, respectively. These results are better than the mAP of 70.08, 63.61 and 66.41 obtained by using the PRS-derived model, respectively, in the KBCP, the OBCS and the merged KBCP-OBCS sample sets. It was also noticed that the identified BC risk-predictive SNPs from the proposed method perform favorably in classifying ER+ and ER− BC cases both in the KBCP and the OBCS sample sets.

One of the challenges of the present study is the lack of suboptimal amount of genotyped BC data to train high-performance BC risk prediction models. To compensate this, we evaluated our proposed as well as the baseline methods in 10 repetitions of 5-fold CV. This iterative partitioning placed the genotyped data of the BC patients and controls in various non-overlapping training, validation and test folds, and consequently resulted in identification of the corresponding BC risk-predictive SNPs, which accounted for possible heterogeneity among BC cases. Further, our biological gene interaction analysis revealed and validated the role of the identified interacting SNPs in important BC related mechanisms, such as estrogen metabolism and apoptosis.

The generalization capability of the proposed method is limited as the prediction performance is lower when the OBCS data is tested with the KBCP identified SNPs and vice versa. However, this might be partly explained by the population-specific variation between the KBCP (Kuopio population) and the OBCS (Oulu population) data and the low-sample size datasets. Note that to get a reasonable predictive generalizability, usually massive amounts of genotyped data from different populations are needed to identify a set of interacting SNPs that generalize well for risk prediction in other populations.

Future work is necessary to improve the generalization capability of the proposed method. We plan to investigate the effectiveness of the proposed method and validate our results with an extended dataset. In this study, we have not included any other data than genomic variants. In the future, we will test the model with other datasets, such as microRNAs, protein interaction, DNA-sequencing and histopathological data. In specific, we will investigate integrating demographic and epidemiological information to the genotyped data in a BC risk prediction task using deep learning frameworks.

To summarize, the novelties of the present study are as follows (i) identifying group of interacting SNPs, which contribute most to the BC risk by means of machine learning, (ii) taking advantage of non-linear feature selection algorithms by assuming dependencies among the SNPs and between the SNPs and the BC risk, (iii) capturing wide range of SNP-SNP interaction patterns in a BC risk prediction model, (iv) evaluating the BC risk prediction model in an iterative process to compensate the lack of suboptimal amount of genotyped BC data and account for possible heterogeneity among BC cases, and (v) evaluating the biological interaction of the identified combination of SNPs and also their relevance to BC subtypes.

## Electronic supplementary material

Supplementary PDF file

## Data Availability

The datasets generated during and/or analyzed during the current study are available from the corresponding author on reasonable request.
