# Supplementary material for: Machine learning identifies interacting genetic variants contributing to breast cancer risk: A case study in Finnish cases and controls
Source: Sci Rep. 2018 Sep 3;8:13149. doi: 10.1038/s41598-018-31573-5 (PMC6120908; doi:10.1038/s41598-018-31573-5)
Supplement: Supplementary file 1 — Supplementary PDF file [file 41598_2018_31573_MOESM1_ESM.pdf]

Machine learning identifies interacting genetic variants contributing to breast cancer risk: A case study in Finnish cases and controls

Hamid Behravan[1*], Jaana M. Hartikainen[1], Maria Tengström[2,3], Katri Pylkäs[4], Robert Winqvist[4], Veli–Matti Kosma[1,5], and Arto Mannermaa[1,5]

[1]Institute of Clinical Medicine, Pathology and Forensic Medicine, University of Eastern Finland, P.O. Box 1627, FI-70211 Kuopio, Finland.

[2]Institute of Clinical Medicine, Oncology, University of Eastern Finland, P.O. Box 1627, FI-70211 Kuopio, Finland.

[3]Cancer Center, Kuopio University Hospital, Kuopio, P.O. Box 100, FI-70029 Kuopio, Finland.

[4]Laboratory of Cancer Genetics and Tumor Biology, Cancer and Translational Medicine Research Unit and Biocenter Oulu, Northern Finland Laboratory Centre Nordlab Oulu, University of Oulu, Oulu, Finland.

[5]Biobank of Eastern Finland and Central Administration, Kuopio University Hospital, Kuopio, Finland.

[*]corresponding author: hamidbeh@uef.fi

Gradient tree boosting

Boosting is an effective ensemble learning algorithm in which weak classifiers are added sequentially to correct the errors made by existing classifiers towards building a strong classifier. For a given dataset with m features and n samples $D = \{\mathrm{x}_i, y_i\}$ ($|D| = n,\ \mathrm{x}_i \in \mathrm{IR}^{\mathrm{m}},\ y_i \in \mathrm{IR}$), a tree ensemble model uses K additive trees to predict the output as follows[1]:

$$\hat{y}_i = \sum_{k=1}^{K} f_k(\mathrm{x}_i),\ f_k \in F, \tag{S1}$$

where, $F = \{f(\mathrm{x}) = \mathrm{w}_{\mathrm{q(x)}}\}(\mathrm{q} : \mathrm{IR}^{\mathrm{m}} \to T, \mathrm{w} \in \mathrm{IR}^{\mathrm{T}})$ is a space of regression or classification trees (also known as CART). Each f_k corresponds to an independent tree structure q and leaf weights w. Here, T is number of leaves in the tree. A given example is classified into the leaves using the decision rules in the trees (given by q) and the final prediction score is computed by summing up the scores in the corresponding leaves (given by w). A set of functions f_k are then learned by minimizing the following regularized learning objective[1]:

$$L = \sum_{i=1}^{n} l(\hat{y}_i, y_i) + \sum_{k=1}^{K} \Omega(f_k), \tag{S2}$$

where, l denotes a differentiable convex loss function measuring the difference between the target y_i and the prediction $\hat{y}_i$. In this study, we use logistic loss (log-loss) for binary classification to calculate the classification loss as follows[2]:

$$l(\hat{y}_i, y_i) = -y_i \log \hat{y}_i - (1 - y_i) \log (1 - \hat{y}_i) \tag{S3}$$

The second term Ω in equation (S2) represents the regularization of the complexity of the tree functions defined as[1]:

$$\Omega = \gamma T + \frac{1}{2}\lambda \sum_{j=1}^{T} \mathrm{w}_j^2. \tag{S4}$$

Here, λ is the L2 regularization term on weights and γ denotes penalization cost on the number of leaves in a tree.

The learning objective in equation (S2) includes functions as parameters and cannot be optimized in a Euclidean space using conventional optimization techniques. Instead, the gradient tree boosting model is trained in an additive manner, where the prediction of i-th example at the t-th iteration is computed as[1] $\hat{y}_i^{(t)} = \hat{y}_i^{(t-1)} + f_t(\mathrm{x}_i)$. The learning objective at the t-th iteration is then defined as[1]:

$$L^{(t)} = \sum_{i=1}^{n} l(\hat{y}_i^{(t-1)}, y_i + f_t(\mathrm{x}_i)) + \Omega(f_t). \tag{S5}$$

XGBoost uses second order Taylor expansion of the loss function to approximate equation (S5) as follows[1]:

$$L^{(t)} \simeq \hat{L}^{(t)} = \sum_{i=1}^{n} [l(\hat{y}_i^{(t-1)}, y_i) + g_i f_t(\mathrm{x}_i) + \frac{1}{2} h_i f_t^2(\mathrm{x}_i)] + \Omega(f_t), \tag{S6}$$

where, g_i and h_i are the first and the second order gradient statistics on the loss function. After removing constant terms and expanding Ω, equation (S5) can be further simplified as:

$$\hat{L}^{(t)} = \sum_{j=1}^{T} \big[(\sum_{i \in \mathrm{I}_j} g_i)\mathrm{w}_j + \frac{1}{2}(\sum_{i \in \mathrm{I}_j} h_i + \lambda)\mathrm{w}_j^2\big] + \gamma T, \tag{S7}$$

where, $\mathrm{I}_j = \{i | q(\mathrm{x}_i) = j\}$ is the set of indices of instances assigned to the j-th leaf. For a given tree structure $q(\mathrm{x})$, the optimal weight w_j^* of leaf j is computed as[1]:

$$\mathrm{w}_j^* = -\frac{\sum_{i \in \mathrm{I}_j} g_i}{\sum_{i \in \mathrm{I}_j} h_i + \lambda}. \tag{S8}$$

By substituting w_j^* into equation (S7), the resulting objective value is rewritten as[1]:

$$\hat{L}^{(t)}(q) = -\frac{1}{2} \sum_{j=1}^{T} \frac{(\sum_{i \in \mathrm{I}_j} g_i)^2}{\sum_{i \in \mathrm{I}_j} h_i + \lambda} + \gamma T. \tag{S9}$$

The objective in equation (S9) is used as scoring function to evaluate the quality of a tree structure $q(\mathrm{x})$ for classification. This score resembles the impurity measure for evaluating decision trees, except that it also considers model complexity. Since, there can be infinite possible tree structures q, a greedy algorithm that starts from a single leaf and iteratively add branches to the tree is used to grow the tree structure[1]. The loss reduction after a split is used to evaluate the split candidates and is computed as[1]:

$$L_{\text{split}} = \frac{1}{2}\left[\frac{(\sum_{i\in \mathrm{I_L}} g_i)^2}{\sum_{i\in \mathrm{I_L}} h_i + \lambda} + \frac{(\sum_{i\in \mathrm{I_R}} g_i)^2}{\sum_{i\in \mathrm{I_R}} h_i + \lambda} - \frac{(\sum_{i\in \mathrm{I}} g_i)^2}{\sum_{i\in \mathrm{I}} h_i + \lambda}\right] - \gamma \tag{S10}$$

where, $\mathrm{I_L}$ and $\mathrm{I_R}$ are the instance sets of left and right nodes after the split and $\mathrm{I} = \mathrm{I_L} \cup \mathrm{I_R}$.

For a single tree f, the relative importance score of each predictor variable x_r (SNPs in this study) is estimated as[3]:

$$\Delta_r^2(f) = \sum_{p=1}^{P-1} \hat{\tau}_p^2. \tag{S11}$$

Each node p partitions the region associated with that into two sub-regions using splitting variable x_r associated with the node. Here, the summation is over the non-terminal nodes p of the P internal nodes of the tree, and $\hat{\tau}_p^2$ denotes the corresponding empirical improvement in squared error as a result of the split[4]. The squared relative importance of variable x_r is the sum of such improvements in squared error over all non-terminal nodes for which it was selected as the splitting variable. The feature importances are then averaged across all of the trees within the model as[3]:

$$\Delta_r^2 = \frac{1}{K}\sum_{k=1}^{K} \Delta_r^2(f_k). \tag{S12}$$

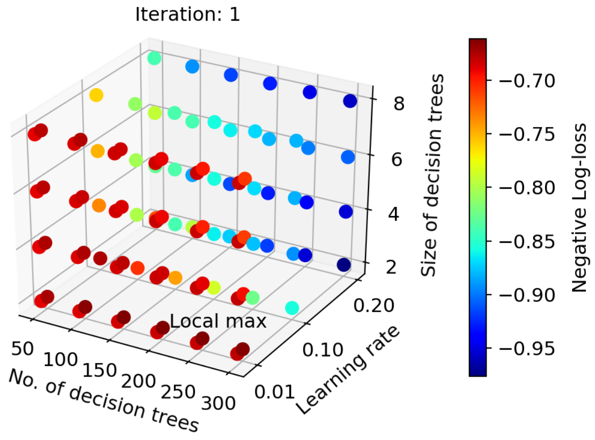
Iteration: 1
Local max
No. of decision trees
Learning rate
Size of decision trees
Negative Log-loss

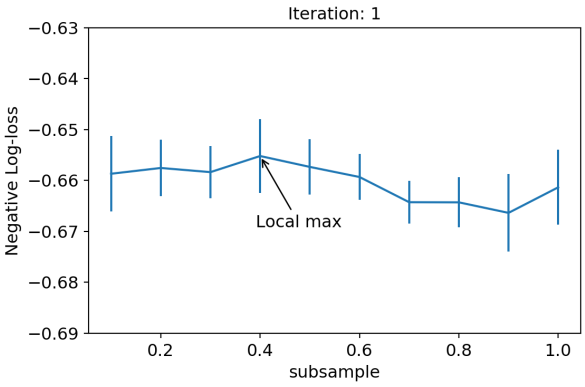
Iteration: 1
Local max
subsample
Negative Log-loss

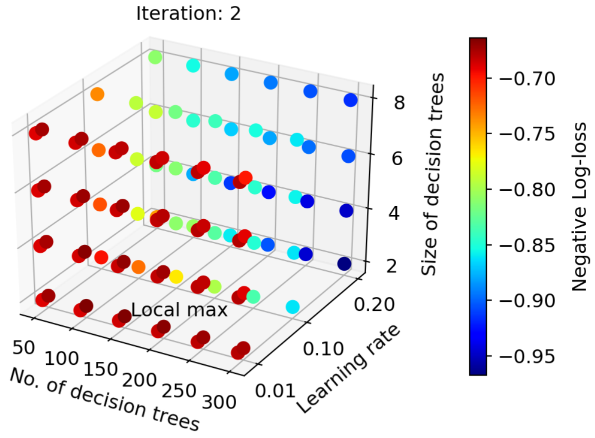
Iteration: 2
Local max
No. of decision trees
Learning rate
Size of decision trees
Negative Log-loss

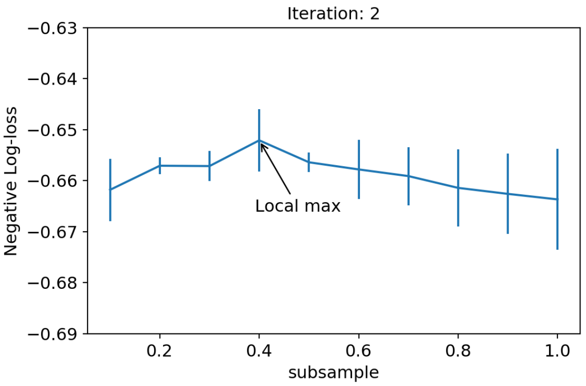
Iteration: 2
Local max
subsample
Negative Log-loss

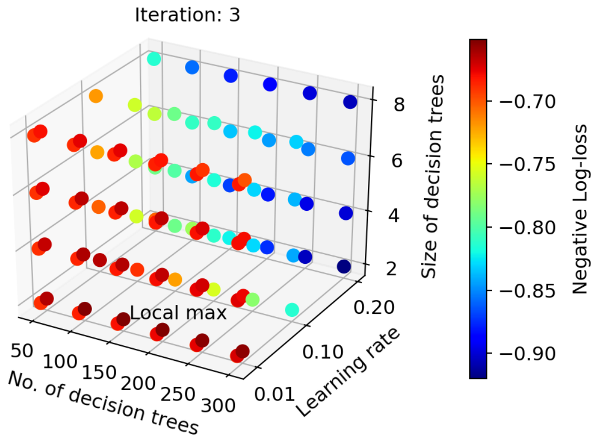
Iteration: 3
Local max
No. of decision trees
Learning rate
Size of decision trees
Negative Log-loss

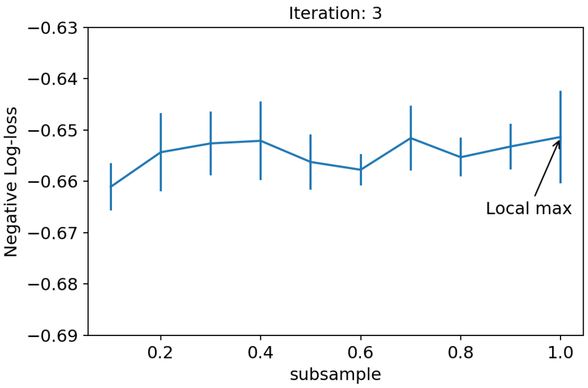
Iteration: 3
Local max
subsample
Negative Log-loss

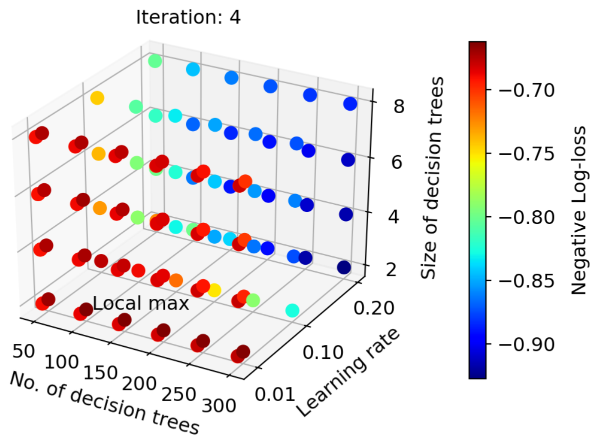
Iteration: 4
Local max
No. of decision trees
Learning rate
Size of decision trees
Negative Log-loss

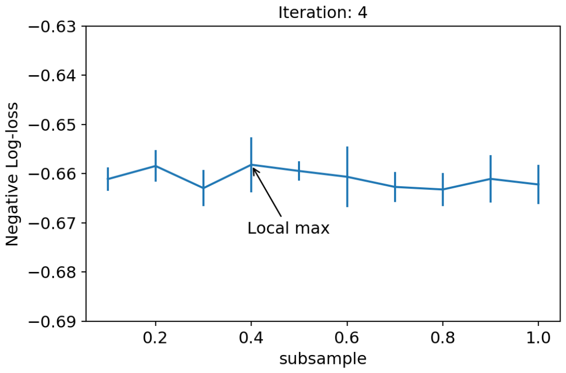
Iteration: 4
Local max
subsample
Negative Log-loss

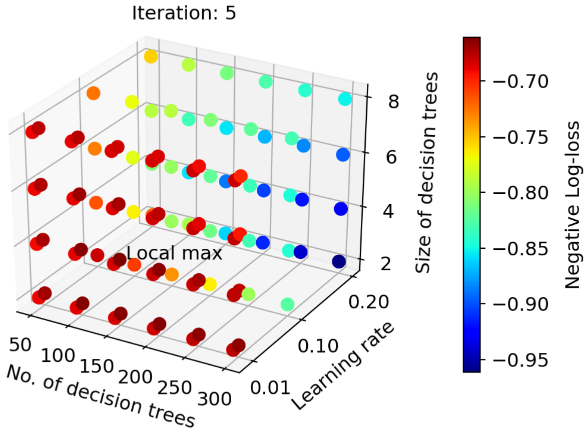
Iteration: 5
Local max
No. of decision trees
50 100 150 200 250 300
Learning rate
0.01 0.10 0.20
Size of decision trees
2 4 6 8
Negative Log-loss
−0.70 −0.75 −0.80 −0.85 −0.90 −0.95

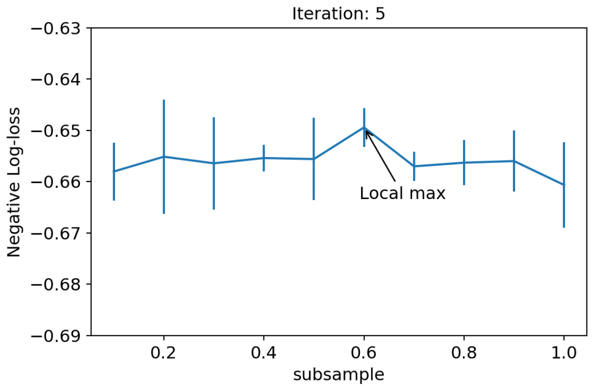
Iteration: 5
Negative Log-loss
−0.63 −0.64 −0.65 −0.66 −0.67 −0.68 −0.69
Local max
subsample
0.2 0.4 0.6 0.8 1.0

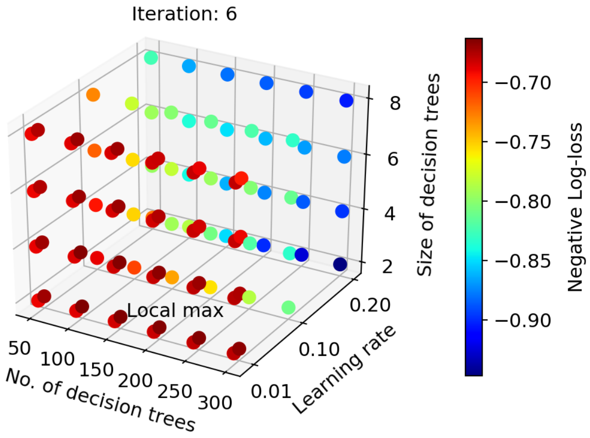
Iteration: 6
Local max
No. of decision trees
50 100 150 200 250 300
Learning rate
0.01 0.10 0.20
Size of decision trees
2 4 6 8
Negative Log-loss
−0.70 −0.75 −0.80 −0.85 −0.90

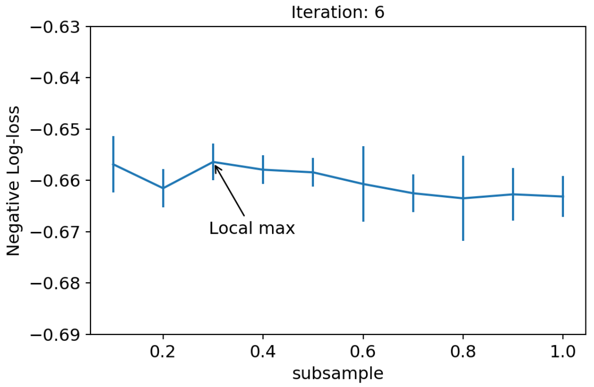
Iteration: 6
Negative Log-loss
−0.63 −0.64 −0.65 −0.66 −0.67 −0.68 −0.69
Local max
subsample
0.2 0.4 0.6 0.8 1.0

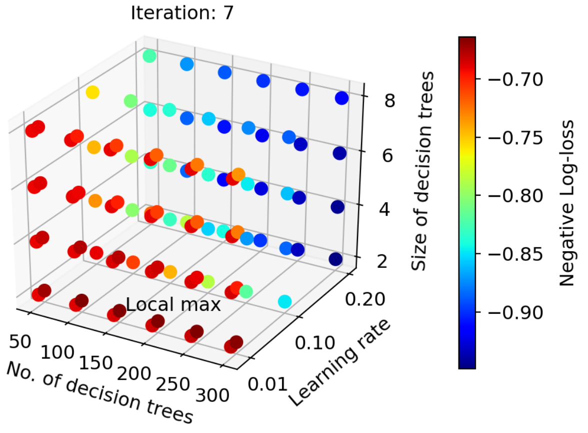
Iteration: 7
Local max
No. of decision trees
50 100 150 200 250 300
Learning rate
0.01 0.10 0.20
Size of decision trees
2 4 6 8
Negative Log-loss
−0.70 −0.75 −0.80 −0.85 −0.90

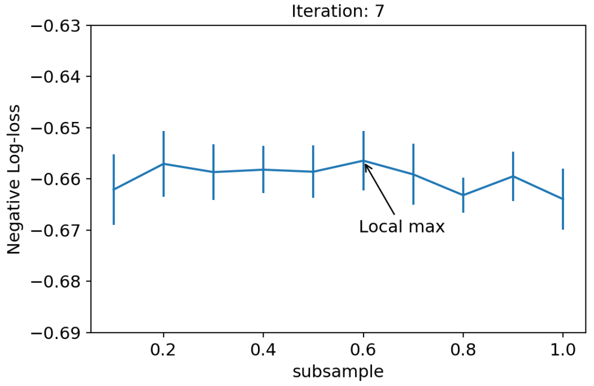
Iteration: 7
Negative Log-loss
−0.63 −0.64 −0.65 −0.66 −0.67 −0.68 −0.69
Local max
subsample
0.2 0.4 0.6 0.8 1.0

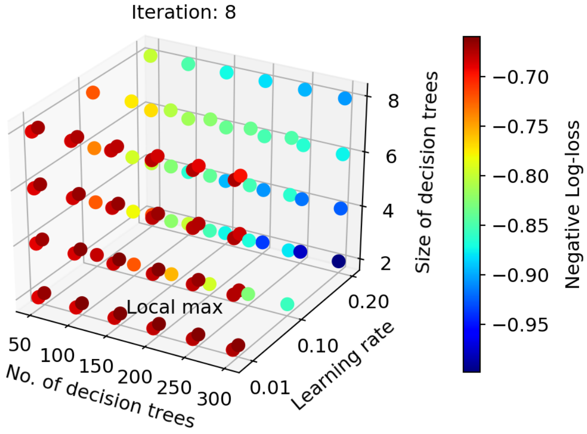
Iteration: 8
Local max
No. of decision trees
50 100 150 200 250 300
Learning rate
0.01 0.10 0.20
Size of decision trees
2 4 6 8
Negative Log-loss
−0.70 −0.75 −0.80 −0.85 −0.90 −0.95

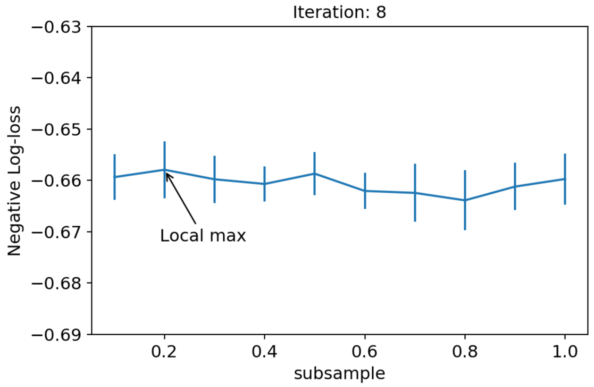
Iteration: 8
Negative Log-loss
−0.63 −0.64 −0.65 −0.66 −0.67 −0.68 −0.69
Local max
subsample
0.2 0.4 0.6 0.8 1.0

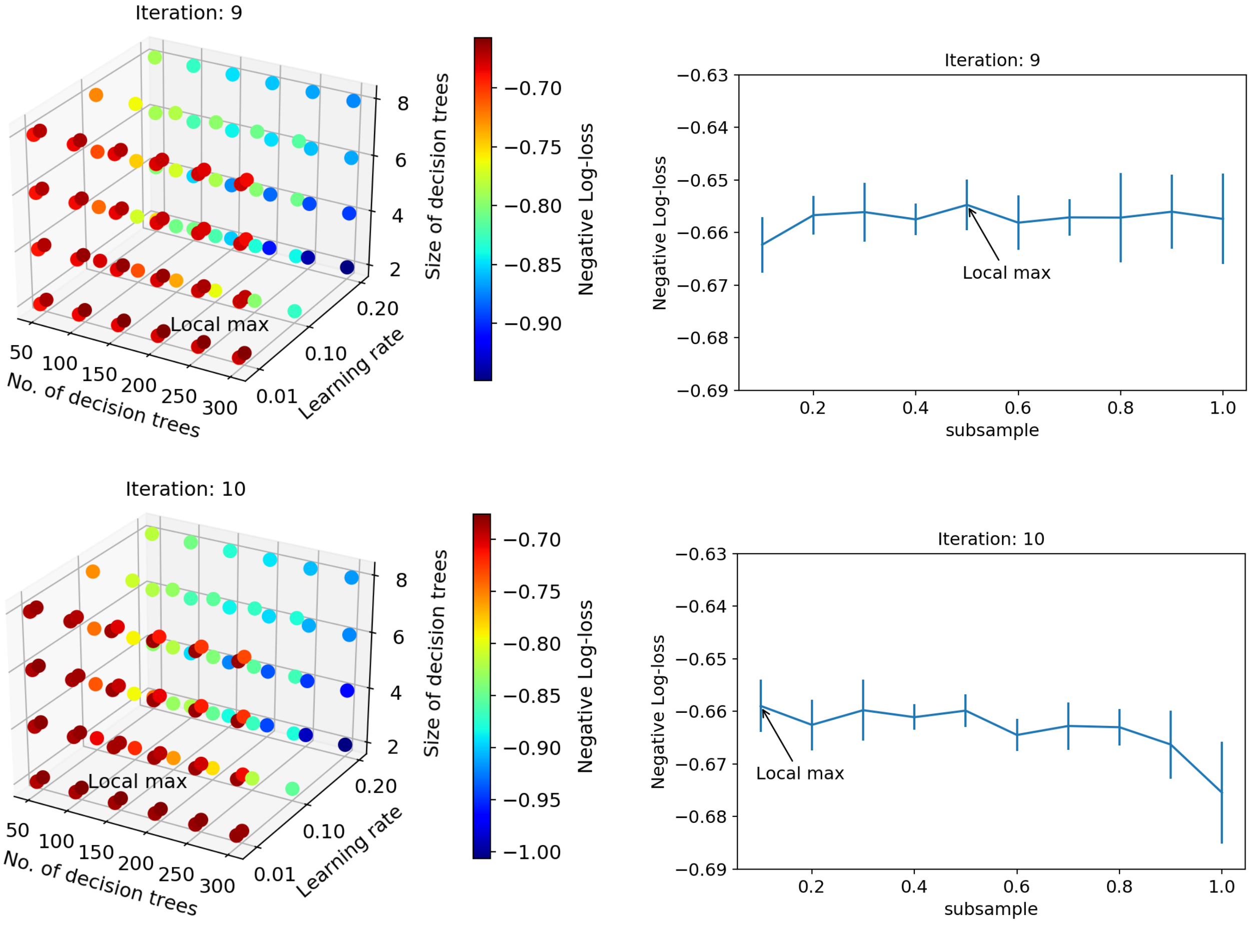

Figure S1. XGBoost hyperparameter optimization. **Left)** Negative log-loss of the model performance as a function of number of decision trees, learning rate and size of decision trees. **Right)** Negative log-loss of model performance as a function of subsampling rate. Local max points to the optimal value of the hyperparameters achieved within each iteration.

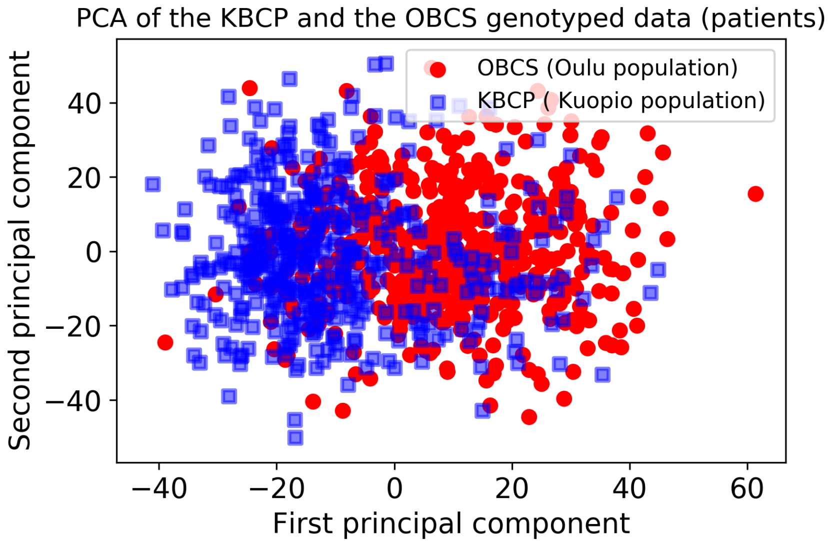

Figure S2. Projection of the KBCP (Kuopio population) and the OBCS (Oulu population) BC cases into a 2D space using a principal component analysis (PCA) method over all available SNPs (#125,041). Each point corresponds to a patient and color codes represent the Kuopio and the Oulu populations. The projection illustrates the population-specific variations between the KBCP and the OBCS genotyped data, which may partly explain the differences between the identified BC risk-predictive SNPs from these two sample sets.

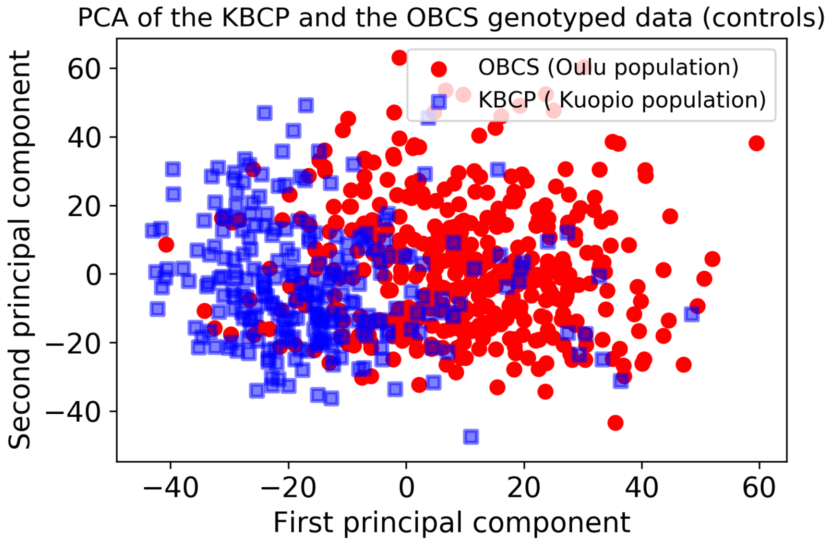

Figure S3. Projection of the KBCP (Kuopio population) and the OBCS (Oulu population) healthy controls into a 2D space using a principal component analysis (PCA) method over all available SNPs (#125,041).

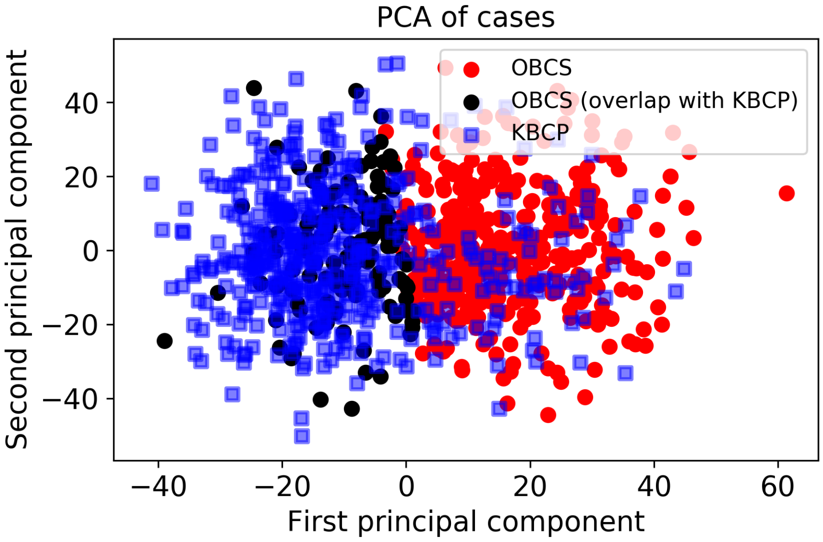

Figure S4. The OBCS cases overlap with the KBCP cases. Black points denote the OBCS cases, which are closely related to the KBCP cases in terms of genetic structure in the PCA space. Combined red and black points represent the original OBCS case data.

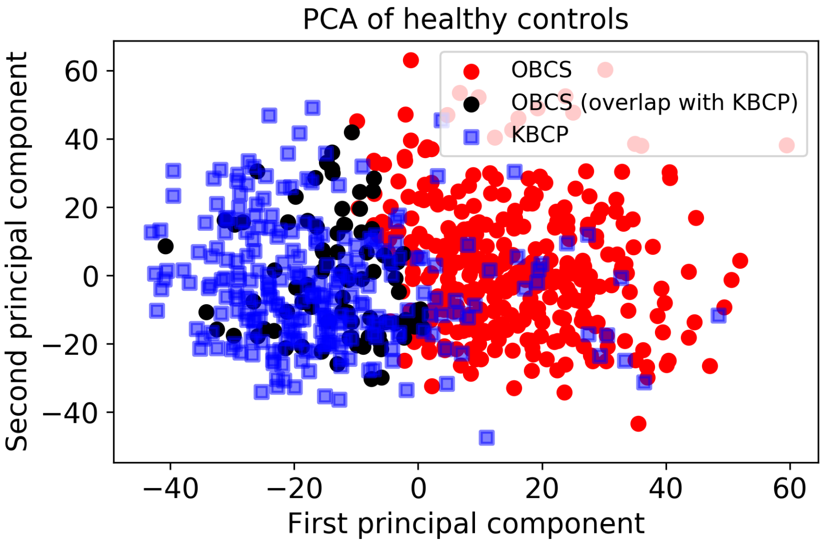

Figure S5. The OBCS controls overlap with the KBCP controls. Black points denote the OBCS controls, which are closely related to the KBCP controls in terms of genetic structure in the PCA space. Combined red and black points represent the original OBCS control data.

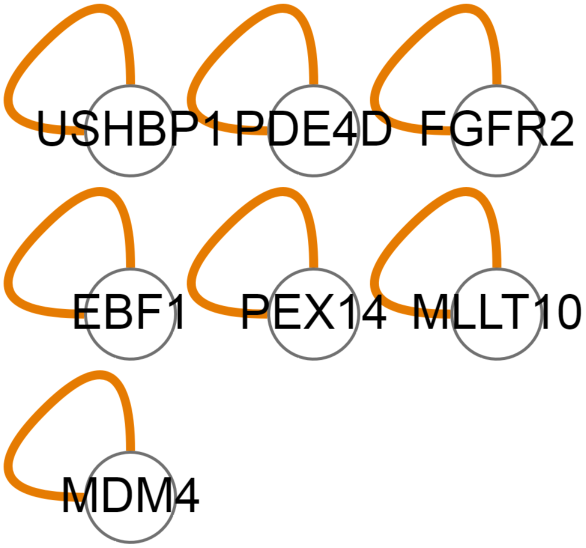

Figure S6. Gene interaction map of the 51 published BC-associated SNPs shows that the literature SNPs form individual entities with no genetic interactions.

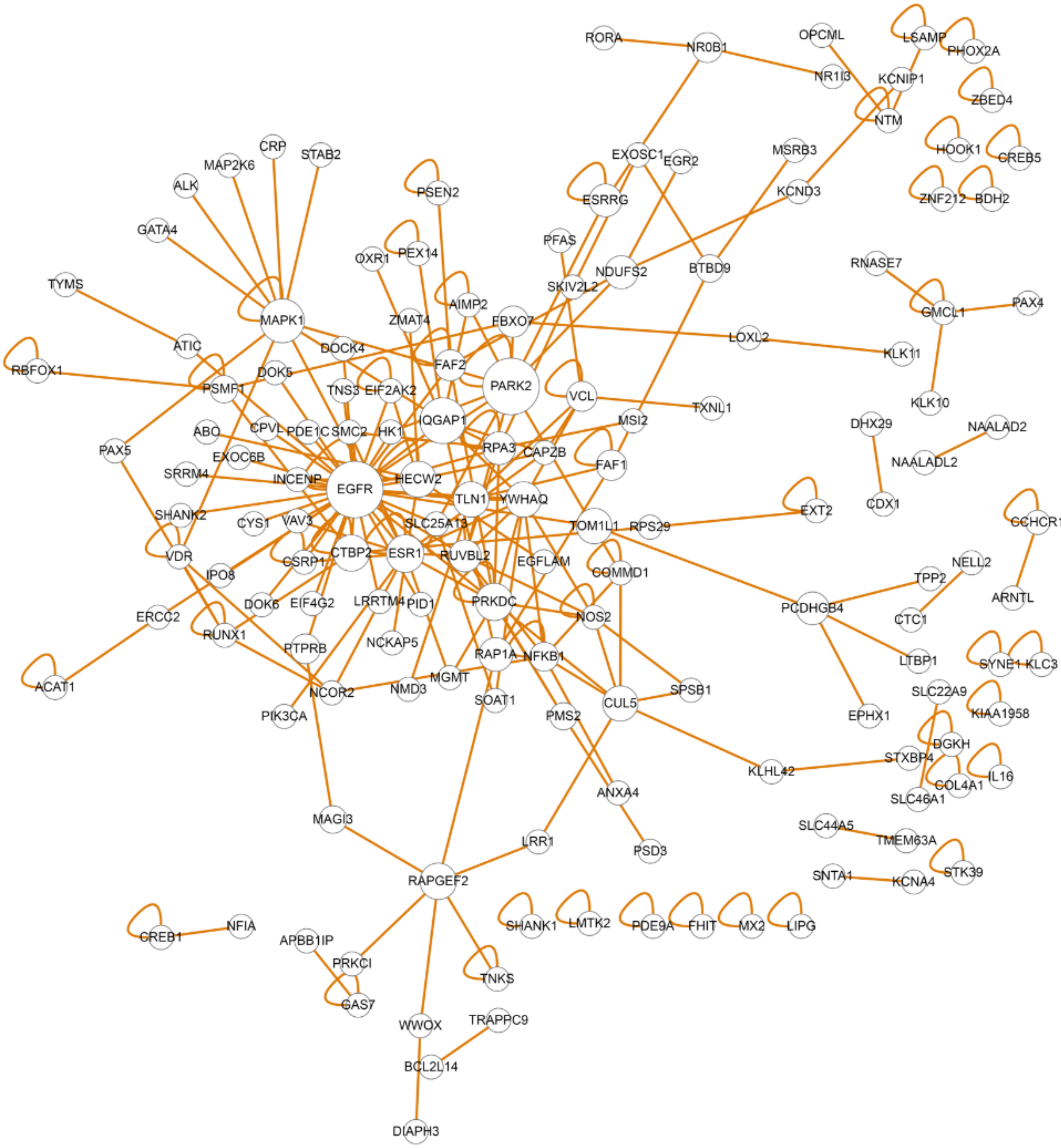

Figure S7. Gene interaction map of the identified OBCS SNPs reveals that the proposed approach can identify interacting genetic variants truly biologically relevant to the BC risk.

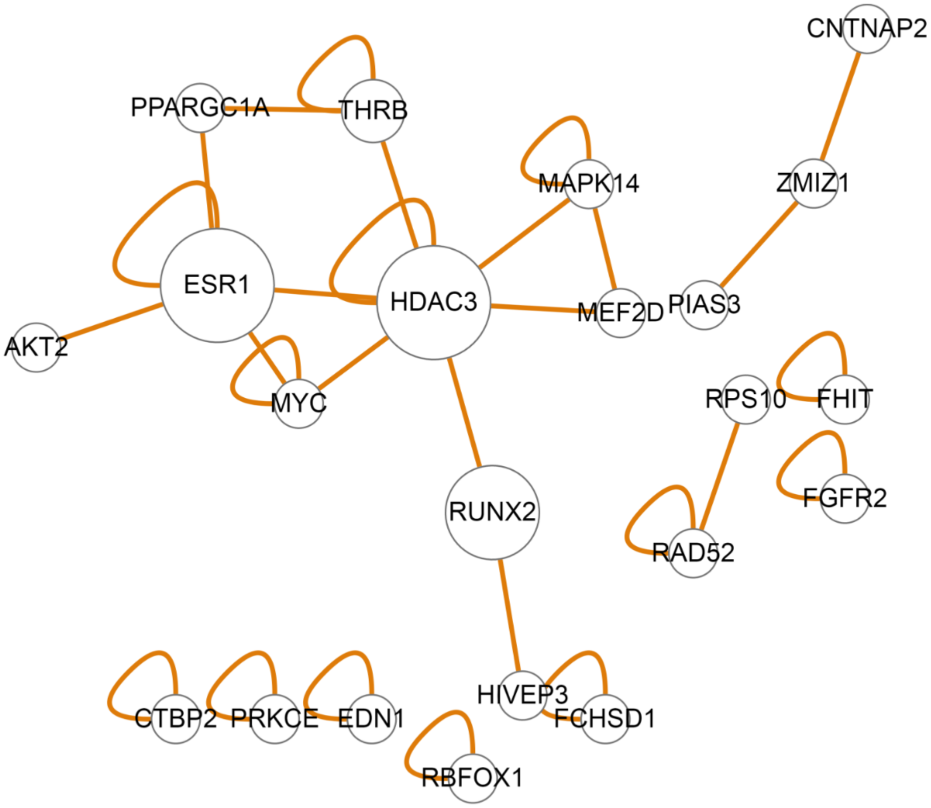

Figure S8. Gene interaction map of the identified SNPs from the merged KBCP and OBCS sample sets reveals that the proposed approach can identify interacting genetic variants truly biologically relevant to the BC risk.

SNP name	Odd ratio	Effect allele
rs616488*	1.1	A
rs4245739	1.03	C
rs12710696	1.04	A
rs4849887*	1.1	G
rs1550623*	1.06	A
rs6762644	1.07	G
rs1053338	1.08	G
rs7726159	1.04	A
rs2736108	1.07	G
rs889312	1.12	C
rs10472076	1.05	G
rs1353747*	1.09	A
rs1432679	1.07	G
rs11242675*	1.06	A
rs17529111	1.06	G
rs12662670	1.17	C
rs2046210	1.08	A
rs9693444	1.07	A
rs11780156	1.07	A
rs1011970	1.06	A
rs10759243	1.05	A
rs865686*	1.12	A
rs7072776	1.07	A
rs11814448	1.27	C
rs704010	1.08	A
rs7904519	1.05	G
rs11199914*	1.05	G
rs2981579	1.27	A
rs3817198	1.07	G
rs554219	1.26	G
rs11820646*	1.05	G
rs12422552	1.04	C
rs10771399*	1.16	A
rs17356907*	1.1	A
rs1292011*	1.09	A
rs11571833	1.27	T
rs2588809	1.08	A
rs999737*	1.09	G
rs941764	1.07	G
rs3803662	1.24	A
rs17817449*	1.09	A
rs11075995	1.04	A
rs13329835	1.08	G
rs6504950*	1.06	G
rs527616*	1.05	G
rs1436904*	1.04	A
rs2363956	1.03	A
rs4808801*	1.08	A
rs2823093*	1.09	G
rs132390	1.14	G
rs6001930	1.12	G

* OR value and effect allele derived from GWAS Central (http://www.gwascentral.org/).

Table S1. List of 51 published BC-associated SNPs and their corresponding odd ratio (OR) value from[5].

Parameters	No. of decision trees	Size of decision trees	Learning rate	Subsampling rate
**Search range**	**[50, 100, 150, 200, 250, 300]**	**[2, 4, 6, 8]**	**[0.001, 0.01, 0.1]**	**[0.1, 0.2, 0.3, 0.4, 0.5, 0.6, 0.7, 0.8, 0.9, 1]**
1	200	2	0.01	0.4
2	150	2	0.01	0.4
3	150	2	0.01	1
4	100	2	0.01	0.4
5	150	4	0.01	0.6
6	150	2	0.01	0.3
7	150	2	0.01	0.6
8	150	2	0.01	0.2
9	200	2	0.01	0.5
10	100	2	0.01	0.1

Table S2. A summary of the optimal XGBoost hyperparameter values achieved within each iteration.

Split round	SNP window size	Adaptive window size increase	Average precision
1	2	1	78.95461827
2	20	4	75.49345761
3	30	3	80.47742845
4	2	2	76.18107292
5	6	3	76.86609958
6	8	4	74.93817069
7	2	5	81.43689464
8	8	5	80.31233859
9	2	1	79.44159918
10	8	5	80.11265793
11	2	1	72.90368555
12	2	1	77.978596
13	4	4	81.78834779
14	10	4	78.59926249
15	8	3	76.93767711
16	2	1	74.02004239
17	2	5	76.07461188
18	30	1	75.99442136
19	4	2	79.23308432
20	10	5	81.0145353
21	4	4	76.99239083
22	10	2	79.10163685
23	20	1	80.58867398
24	2	1	74.69651416
25	2	5	76.93875729
26	20	3	81.52875587
27	20	4	76.38343732
28	8	4	77.28456672
29	20	1	75.81992233
30	8	5	81.82803765
31	6	3	77.01280357
32	4	2	79.86267831
33	4	3	80.51885978
34	10	4	75.17447939
35	20	5	80.03074865
36	2	1	80.0804829
37	8	2	77.54879698
38	2	1	74.09519107
39	30	3	80.11827962
40	4	1	77.74499298
41	8	3	79.76069879
42	2	1	77.13881487
43	10	4	77.36499161
44	2	1	79.7628486
45	2	1	77.42541225
46	4	2	77.53744874
47	4	4	76.34506576
48	2	5	80.17550905
49	6	5	80.17550905
50	2	2	76.72200912

Table S3. The optimal value of SNP window size and the adaptive window size increase found within each train/validation split round in the 10 iterations of 5-fold CV. Smaller SNP window sizes are often marked as optimal values. SNP window size = 2 is found optimal in 17 out of 50 splits. Similarly, the adaptive window size increase = 1 is found optimal in 15 out of 50 splits.

Table S4. Identified interacting SNPs from the KBCP sample set and their associated genes.

Variant name	Chromosome	Position start (bp)	Associated gene name
rs10042283	5	15915052	FBXL7
rs10081395	7	69046227	AC104688.1
rs10083612	15	32668424	AC123768.1
rs10083612	15	32668424	AC254945.2
rs10083612	15	32668424	SCG5
rs10088936	8	17379961	MTMR7
rs1014664	6	155275566	CLDN20
rs1014664	6	155275566	TFB1M
rs10148270	14	85500620	AL049775.2
rs10157819	1	5620512	AL365255.1
rs1016343	8	127081052	CASC19
rs1016343	8	127081052	PRNCR1
rs10171836	2	227130112	COL4A4
rs1023294	9	117225627	ASTN2
rs10235235	7	99478208	ZNF789
rs10240660	7	3172446	AC073316.1
rs10274783	7	89312630	ZNF804B
rs1035085	7	13263576	AC011287.1
rs1042779	3	52786995	ITIH1
rs10487888	7	140799307	BRAF
rs10489880	3	197234315	DLG1
rs10502616	18	34016312	NOL4
rs10504034	8	40674623	ZMAT4
rs10510790	3	57162356	IL17RD
rs10510966	3	67681962	SUCLG2-AS1
rs10515638	5	149772968	PPARGC1B
rs10745895	12	100787787	ANO4
rs10782333	6	154460963	CNKSR3
rs10817733	9	115236876	DEC1
rs10847525	12	128086503	LINC02369
rs10848321	12	131237976	AC126564.1
rs10886957	10	121619564	AC009988.1
rs10937751	4	6710329	MRFAP1L1
rs10958704	8	38470784	FGFR1
rs10981863	9	113654025	AL157702.2
rs10991109	9	104102746	SMC2
rs11022395	11	12531405	PARVA
rs11039856	11	5952264	OR56A3
rs11056575	12	15595481	PTPRO
rs11075994	16	53816167	FTO
rs11089838	22	37386898	ELFN2
rs11109055	12	97478629	RMST
rs11127264	2	30284583	LBH
rs11144870	9	76389297	RFK
rs11255182	10	7530902	AL445070.1
rs11258998	10	14294634	FRMD4A
rs1132185	1	12760937	C1orf158
rs11643994	16	9402803	LINC02177
rs11649879	17	78443027	DNAH17
rs11684027	2	223200663	KCNE4
rs11703324	22	40335130	TNRC6B
rs11769484	7	27881667	JAZF1

rs11845605	14	100571389	BEGAIN
rs11850085	14	70086846	SLC8A3
rs11869485	17	3497942	ASPA
rs11869485	17	3497942	SPATA22
rs11919311	3	49619356	BSN
rs1206988	6	10024574	OFCC1
rs12145453	1	30040878	LINC01648
rs12210447	6	42748696	BICRAL
rs12210447	6	42748696	TBCC
rs12224675	11	11985530	DKK3
rs12257319	10	12789103	CAMK1D
rs12310676	12	69716927	AC025263.1
rs1245120	11	113130674	NCAM1
rs12468506	2	43334280	THADA
rs12641904	4	11364392	MIR572
rs12876308	13	110429327	AL159153.1
rs12876308	13	110429327	COL4A2
rs13082560	3	167329933	ZBBX
rs13179555	5	76152369	SV2C
rs13396251	2	1991119	MYT1L
rs13407676	2	12232846	MIR3681HG
rs13415994	2	12978394	AC064875.1
rs1343921	4	89026311	FAM13A
rs1367088	8	27404678	PTK2B
rs1370216	18	57021585	WDR7
rs137963	22	39907930	GRAP2
rs1403247	11	14611024	PSMA1
rs1424885	14	44781215	LINC02302
rs1468052	6	133688556	TARID
rs1470811	15	86814575	AGBL1
rs1472874	12	24422338	AC069208.1
rs1475891	21	43154431	AP001631.1
rs1491267	4	13701457	LINC01182
rs1503128	3	76499267	ROBO2
rs1522527	7	96855293	MARK2P10
rs1528431	2	150489863	RND3
rs153706	5	16882133	MYO10
rs1552844	17	72944841	SLC39A11
rs1579303	5	181068340	AC008620.1
rs1595455	2	153534267	AC012501.1
rs162899	5	132279900	P4HA2
rs163077	2	38058462	RMDN2
rs163077	2	38058462	RMDN2-AS1
rs164815	5	77744405	TBCA
rs16823635	2	180709348	SCHLAP1
rs16846848	1	227031285	CDC42BPA
rs16907395	12	11844686	ETV6
rs17008611	2	73034721	SFXN5
rs17012668	2	33209915	LTBP1
rs17021211	1	104204629	AC092506.1
rs17023386	1	119293520	AL359915.1
rs17068808	3	63489618	SYNPR
rs17068808	3	63489618	SYNPR-AS1
rs1726595	7	18585909	HDAC9
rs17271883	19	5834201	FUT6

rs1727353	12	131099862	ADGRD1
rs17330637		123909855	XIAP
rs17363184	19	4635390	TNFAIP8L1
rs17623404	20	15653163	MACROD2
rs17751951	2	18553665	NT5C1B-RDH14
rs17751951	2	18553665	RDH14
rs17847528	17	37729021	HNF1B
rs181410	22	17754670	BID
rs1874835	15	27799154	OCA2
rs1897558	5	168125600	AC011369.1
rs1897558	5	168125600	AC011369.2
rs1897558	5	168125600	TENM2
rs1944937	11	74708312	CHRDL2
rs1950771	14	94798565	AL121612.1
rs1956193	14	59017785	LINC01500
rs197105	1	57211646	DAB1
rs1999805	6	151747229	ESR1
rs2006779	20	18808585	C20orf78
rs2007873	8	61111425	CLVS1
rs2028900	2	85540612	GGCX
rs2028900	2	85540612	MAT2A
rs2034537	4	65378651	EPHA5
rs2047169	2	237714770	LRRFIP1
rs2059273	16	6001142	RBFOX1
rs2073204	14	30889171	AL049830.3
rs2073204	14	30889171	COCH
rs2073204	14	30889171	STRN3
rs2078276	10	95327302	SORBS1
rs2084872	2	208105141	CRYGEP
rs2098469	12	13918072	GRIN2B
rs2098683	19	50560422	AC008743.1
rs2098683	19	50560422	LRRC4B
rs2113616	2	86307397	REEP1
rs2157088	22	33815448	LARGE1
rs2168681	4	182422410	TENM3
rs2181502	13	99331065	UBAC2
rs220370	10	24299314	KIAA1217
rs2212763	11	86924920	AP001528.2
rs2212763	11	86924920	PRSS23
rs2216188	16	52494272	TOX3
rs2255831	4	145335556	LINC02266
rs2268084	20	34043582	RALY
rs2273026	17	18353665	SHMT1
rs2283318	12	2501475	CACNA1C
rs2325514	13	73438653	LINC00393
rs2409691	8	11085766	XKR6
rs2448050		12096290	BCL2L14
rs2458286	8	102966471	AZIN1-AS1
rs2542970	18	45427721	SLC14A2
rs2542970	18	45427721	SLC14A2-AS1
rs2548656	5	95397446	FAM81B
rs2555236	3	37565477	ITGA9
rs2607831	10	86209725	GRID1
rs2608897	6	131572323	ARG1
rs2608897	6	131572323	MED23

rs2643154	12	45072561	AC008127.1
rs2643154	12	45072561	AC008127.2
rs265598	12	129655961	TMEM132D
rs2717756	8	18572153	PSD3
rs2723441	7	13482316	AC011287.1
rs2764664	1	57275490	DAB1
rs2813538	6	152200109	SYNE1
rs2817174	1	3127617	MIR4251
rs2817174	1	3127617	PRDM16
rs2837461	21	40150682	DSCAM
rs2859420	6	35835506	LHFPL5
rs2859420	6	35835506	SRPK1
rs2866016	4	98583084	TSPAN5
rs2908964	17	11539340	SHISA6
rs294218	1	22706764	EPHB2
rs3027001	1	159199673	ACKR1
rs3027001	1	159199673	CADM3
rs3027001	1	159199673	CADM3-AS1
rs3107475	8	103757554	RIMS2
rs3133861	11	120788776	GRIK4
rs3217992	9	22003224	AL359922.1
rs3217992	9	22003224	CDKN2B
rs3217992	9	22003224	CDKN2B-AS1
rs3734165	5	138325745	CDC25C
rs3742723	14	78809235	NRXN3
rs3753535	1	178867836	ANGPTL1
rs3753535	1	178867836	RALGPS2
rs3770120	2	181487742	ITGA4
rs3787138	20	63347872	CHRNA4
rs3787268	20	46013092	MMP9
rs3787268	20	46013092	SLC12A5-AS1
rs3803371	15	50197861	SLC27A2
rs3809977	18	58536666	AC105105.1
rs3809977	18	58536666	ALPK2
rs3810027	18	63236745	BCL2
rs389686	5	69159339	SNORA50D
rs4068867	2	68748722	ARHGAP25
rs4072263	5	174820431	AC113346.1
rs4072263	5	174820431	AC113346.2
rs4077952	10	126419412	LINC00601
rs4135063	12	103971884	TDG
rs4147101	10	110779004	RBM20
rs415704	17	7074375	CLEC10A
rs432717	20	63599621	GMEB2
rs4348044	4	4851140	LINC01396
rs4456611	18	63143219	BCL2
rs4543420	6	6383915	LY86-AS1
rs4627704	3	73822774	LINC02005
rs4729090	7	93428291	CALCR
rs4746864	10	69581444	AL450311.2
rs4778603	15	79028117	RASGRF1
rs478859	1	100330564	AC104457.1
rs479193	11	107559574	ALKBH8
rs479772	10	82865171	NRG3
rs4810648	20	47639206	NCOA3

rs4872139	8	23489128	AC104561.1
rs4872139	8	23489128	AC104561.3
rs487637	6	151018306	MTHFD1L
rs4880616	10	3507174	AL357833.1
rs4969187	17	78409756	PGS1
rs4996007	2	239934642	NDUFA10
rs5012899	7	11206526	AC004160.1
rs510411	4	56290213	KIAA1211
rs510411	4	56290213	RNU6-197P
rs524554	3	140470306	CLSTN2
rs5750508	22	37907148	MICALL1
rs5750547	22	38150693	AL022322.2
rs5750547	22	38150693	PLA2G6
rs581886	1	57301423	DAB1
rs584383	1	57239533	DAB1
rs5927633		30871740	TAB3
rs5970645		22797590	PTCHD1-AS
rs6001209	22	38725247	GTPBP1
rs6001209	22	38725247	PRDX3P1
rs6105103	20	13447589	TASP1
rs640706	9	22687164	LINC01239
rs6438300	3	116203164	LSAMP
rs6442199	3	10682201	ATP2B2
rs6450652	5	28621186	RNU6-909P
rs6451229	5	35866116	IL7R
rs6513412	20	58787469	PIEZO1P2
rs6553017	4	186660322	FAT1
rs6716551	2	43708586	PLEKHH2
rs6751507	2	124627096	CNTNAP5
rs6770985	3	63020917	LINC00698
rs6787941	3	159369876	IQCJ-SCHIP1
rs6816024		155946329	CTSO
rs6824447	4	110199557	ELOVL6
rs686699	11	31864033	RCN1
rs6964466	7	19098234	TWIST1
rs6994582	8	68139316	PREX2
rs704617	11	29629752	LINC02546
rs7075753	10	127553356	NPS
rs7104359	11	103826689	AP002989.1
rs7135	16	19503161	AC130456.5
rs7135	16	19503161	AC130456.6
rs7135	16	19503161	GDE1
rs7135	16	19503161	RNU4-46P
rs7135	16	19503161	TMC5
rs7252788	19	9233179	OR7D1P
rs735538	19	5308033	PTPRS
rs743562	5	132536691	AC116366.3
rs743562	5	132536691	IL5
rs7508	8	18056461	ASAH1
rs7525637	1	119000848	AL139420.1
rs7525637	1	119000848	AL139420.2
rs7552664	1	12217910	RPL10P17
rs7552664	1	12217910	SNORA70
rs757158	7	95326216	PON1
rs7597072	2	229495371	DNER

rs7601227	2	174014446	AC016737.2
rs7648642	3	119542528	CD80
rs7650433	3	40857673	AC099560.1
rs7689823	4	186188712	CYP4V2
rs7689823	4	186188712	FLJ38576
rs7712739	5	125332755	AC109471.2
rs7712739	5	125332755	AC109471.3
rs7764938	6	143899267	AL049844.3
rs7764938	6	143899267	ZC2HC1B
rs7901640	10	18368024	CACNB2
rs7995765	13	102177067	FGF14
rs7996852	13	49480210	SETDB2
rs807573	2	15667513	AC008278.1
rs912946	13	110495499	COL4A2
rs915179	1	156108458	LMNA
rs9303690	17	35678494	AP2B1
rs930436	7	154944240	PAXIP1
rs930436	7	154944240	PAXIP1-AS2
rs9309559	2	26918647	DPYSL5
rs9365352	6	161919918	PRKN
rs947661	1	156525124	IQGAP3
rs9600123	13	73413754	LINC00393
rs963447		15535373	BMX
rs9676602	19	49419739	CCDC155
rs9676602	19	49419739	GFY
rs9676602	19	49419739	PTH2
rs981572	17	56071113	ANKFN1
rs9823034	3	184575822	EPHB3
rs9831563	3	193697961	OPA1
rs983474	3	100786744	ABI3BP
rs9844515	3	132835290	NPHP3-AS1
rs9847298	3	195852938	LINC01983
rs9882865	3	6896321	GRM7
rs9882865	3	6896321	GRM7-AS2
rs9884630	4	137212675	LINC02511
rs9962656	18	63180029	BCL2

Table S5. Identified interacting SNPs from the OBCS sample set and their associated genes.

Variant Name	Chromosome	Position start (bp)	Associated gene name
rs10008860	4	7967867	-
rs10008860	4	7967867	ABLIM2
rs10020809	4	79884276	PCAT4
rs10034075	4	46086179	GABRG1
rs10056418	5	63193613	-
rs1009490	18	61466500	CDH20
rs1009668	1	225852703	AL591895.1
rs1009668	1	225852703	EPHX1
rs1009668	1	225852703	TMEM63A
rs1010859	2	38875386	-
rs1010859	2	38875386	DHX57
rs1010859	2	38875386	MORN2
rs1010859	2	38875386	RNU6-851P
rs10132354	14	46816563	-
rs10133734	14	106596292	IGHV3-52
rs10133734	14	106596292	IGHV3-53
rs10133734	14	106596292	IGHV3-54
rs10133734	14	106596292	IGHV4-55
rs10133734	14	106596292	IGHVII-53-1
rs1020120	9	84611173	AL354692.1
rs10202341	2	229045274	PID1
rs10219714	12	18966201	-
rs10251504	7	78060571	MAGI2
rs10253670	7	41439299	-
rs1033805	1	168983296	LINC00970
rs1040637	6	134502781	AL078590.2
rs1040637	6	134502781	LINC01010
rs10413904	19	29084593	-
rs10428295	4	27564890	-
rs10439341	2	209345783	-
rs10440830	6	14026870	-
rs10456575	6	48043832	PTCHD4
rs10458657	10	74021178	-
rs10458657	10	74021178	AL596247.1
rs10458657	10	74021178	VCL
rs10478312	5	116838770	AC010267.1
rs10483172	22	33565906	LARGE1
rs1048328	19	51024108	AC011473.2
rs1048328	19	51024108	AC011473.3
rs1048328	19	51024108	KLK10
rs1048328	19	51024108	KLK11
rs1048328	19	51024108	KLK12
rs10486081	7	13701209	AC011287.1
rs10489628	1	67238424	C1orf141
rs10489628	1	67238424	IL23R
rs10494138	1	111587805	RAP1A
rs1049901	20	1167131	ACTG1P3
rs1049901	20	1167131	PSMF1
rs10502278	11	123902009	OR8D4
rs10504033	8	40633274	ZMAT4
rs10507541	13	46331139	-
rs10509918	10	110471688	-

rs10509918	10	110471688	AL355512.1
rs10515919	2	75313470	-
rs1055461	6	37212267	TMEM217
rs1062708	19	49010016	AC008687.4
rs1062708	19	49010016	LHB
rs1062708	19	49010016	MIR6798
rs1062708	19	49010016	RUVBL2
rs10734146	11	91131160	-
rs10737153	11	69467061	AP000439.4
rs10760188	9	121814788	AL596244.1
rs10760188	9	121814788	TTLL11
rs10761664	10	62847301	-
rs10761664	10	62847301	EGR2
rs10761677	10	62892846	EGR2
rs10770822	12	21529305	GYS2
rs10770822	12	21529305	SPX
rs10773543	12	128439181	AC090424.1
rs10773543	12	128439181	TMEM132C
rs1077971	13	39605151	LHFPL6
rs10791291	11	133413189	OPCML
rs10800777	1	201430960	-
rs10800777	1	201430960	AC096677.3
rs10800777	1	201430960	TNNI1
rs10802960	1	241397750	-
rs10816625	9	108074792	-
rs10838237	11	44130512	EXT2
rs10858627	12	76239248	-
rs10878264	12	65365742	MSRB3
rs10899786	10	43249135	-
rs10899786	10	43249135	RASGEF1A
rs10921244	1	192740808	-
rs10931734	2	196248973	-
rs10931734	2	196248973	HECW2
rs10934131	3	112052101	TMPRSS7
rs10954947	8	34045941	AF279873.3
rs10958605	8	40196086	-
rs10995307	10	62792482	AL133417.1
rs11002768	10	78985343	ZMIZ1-AS1
rs11009247	10	33141481	-
rs11017821	10	131212583	TCERG1L
rs11034562	11	38018790	AC103798.2
rs11061973	12	1756770	ADIPOR2
rs11061973	12	1756770	RPS4XP14
rs11080058	17	28415042	AC015917.2
rs11080058	17	28415042	H3F3BP2
rs11080058	17	28415042	SLC46A1
rs11090767	22	47387702	-
rs1110163	5	178334767	COL23A1
rs11108693	12	96778360	CFAP54
rs11121384	1	9353587	SPSB1
rs11124306	2	33110441	LTBP1
rs11125908	2	62122368	AC018462.1
rs11125908	2	62122368	COMMD1
rs11134316	5	8571938	AC091953.2
rs11134323	5	8691794	AC091953.3

rs11163493	1	75493291	SLC44A5
rs11189224	10	97447069	EXOSC1
rs11189224	10	97447069	ZDHHC16
rs11194526	10	109269026	-
rs11209214	1	68101616	GNG12-AS1
rs11209214	1	68101616	WLS
rs11219453	11	124094306	OR10D4P
rs11224629	11	101202941	-
rs11224629	11	101202941	AP001533.1
rs11224629	11	101202941	PGR-AS1
rs11227014	11	97560300	-
rs1122860	14	84239323	-
rs11236490	11	70514354	SHANK2
rs11264298	1	155036782	DCST1
rs11264298	1	155036782	DCST1-AS1
rs11264298	1	155036782	DCST2
rs11466784	5	157494643	-
rs11466784	5	157494643	ADAM19
rs1149795	1	50506892	FAF1
rs1152187	X	70259092	ARR3
rs1152187	X	70259092	P2RY4
rs1152187	X	70259092	RNA5SP507
rs11563273	2	51623879	AC007402.1
rs11610862	12	19661925	AEBP2
rs11623545	14	71245433	-
rs11630973	15	27251812	GABRG3
rs11632411	15	50144854	ATP8B4
rs11686998	2	41961501	C2orf91
rs11726814	4	103089680	BDH2
rs11726814	4	103089680	SLC9B2
rs11731338	4	57250755	-
rs11741260	5	44411963	FGF10-AS1
rs11746643	5	35833615	AC137810.1
rs11754094	6	105662105	Z97206.1
rs11756073	6	170048356	-
rs11763514	7	117249696	-
rs11815945	10	13221558	RNU6-6P
rs11815945	10	13221558	UCMA
rs11843561	13	26455647	-
rs11850195	14	92860516	-
rs11887670	2	53516972	-
rs11933077	4	155026265	-
rs11944489	4	75055715	PARM1
rs11974621	7	72806109	-
rs11974621	7	72806109	TYW1B
rs1202427	7	149240045	-
rs1202427	7	149240045	ZNF212
rs1205	1	159712443	CRP
rs1205	1	159712443	CRPP1
rs12051691	17	8246469	CTC1
rs12051691	17	8246469	PFAS
rs12066114	1	19466033	-
rs12066114	1	19466033	CAPZB
rs12069337	1	224893795	DNAH14
rs1207955	2	202032326	-

rs1207955	2	202032326	AC069148.1
rs1207955	2	202032326	FZD7
rs12092204	1	69520551	-
rs12137063	1	168283743	TBX19
rs12183158	6	140724219	-
rs12199673	6	73171243	KCNQ5
rs12212637	6	8731898	-
rs12214442	6	98069396	AL589740.1
rs12215821	6	168138002	-
rs12220246	10	30109190	JCAD
rs12356887	10	125094601	CTBP2
rs12358346	10	19469664	MALRD1
rs12402967	1	10557867	PEX14
rs12434808	14	85661455	FLRT2
rs12456229	18	77406456	-
rs12488585	3	1822022	-
rs12577790	11	108115500	-
rs12577790	11	108115500	ACAT1
rs12577790	11	108115500	AP002433.1
rs12577790	11	108115500	CUL5
rs12581304	12	67231721	-
rs12606544	18	49596867	LIPG
rs12611051	19	35167856	FXYD5
rs12624216	2	228374683	-
rs12631850	3	161165274	NMD3
rs12704840	7	96276810	AC096775.1
rs12704840	7	96276810	SLC25A13
rs12758555	1	81922027	ADGRL2
rs1281451	3	59636917	-
rs12815520	12	13333671	-
rs12865465	13	110768612	RPL21P107
rs12907778	15	67842689	AC009292.1
rs12907778	15	67842689	AC009292.2
rs12907778	15	67842689	RNU6-1
rs12907778	15	67842689	SKOR1
rs12915776	15	60787178	RORA
rs12937360	17	54968937	-
rs12937360	17	54968937	AC007485.1
rs12937360	17	54968937	COX11
rs12937360	17	54968937	STXBP4
rs12937360	17	54968937	TOM1L1
rs12968679	18	56597899	TXNL1
rs12971478	19	56923198	AC044792.1
rs130078	6	31150788	CCHCR1
rs130078	6	31150788	POLR2LP1
rs130078	6	31150788	TCF19
rs13020347	2	208651597	PTH2R
rs13074033	3	43175797	-
rs13125117	4	105988541	NPNT
rs13171278	5	176517209	FAF2
rs13171278	5	176517209	RNF44
rs1323120	1	175631043	TNR
rs1325172	10	112598796	VTI1A
rs13256672	8	140028857	TRAPPC9
rs132667	22	36168981	APOL3

rs132667	22	36168981	MTATP6P20
rs132667	22	36168981	MTCO1P20
rs132667	22	36168981	MTCO2P20
rs132667	22	36168981	MTCO3P20
rs132667	22	36168981	MTCYBP34
rs132667	22	36168981	MTND1P10
rs13267369	8	26955643	-
rs13288123	9	36934763	PAX5
rs13295269	9	18537446	ADAMTSL1
rs1330855	1	94295329	AC097059.1
rs1330855	1	94295329	GAPDHP29
rs13417081	2	9629921	-
rs13417081	2	9629921	AC082651.1
rs13417081	2	9629921	YWHAQ
rs1344638	2	204056829	AC009965.2
rs1350092	15	94160857	AC110027.1
rs1367211	6	160661663	AL109933.2
rs1367211	6	160661663	LPA
rs1390902	16	76845072	AC106729.1
rs1391440	4	105195804	TET2
rs1394016	3	114191042	DRD3
rs1407818	1	192592582	-
rs1416527	1	217063459	ESRRG
rs1429751	15	33824657	RYR3
rs1449750	4	27555763	-
rs1450679	9	104264531	TOPORSLP1
rs1451800	3	36013897	-
rs1472848	18	70968191	-
rs1483452	8	93109593	-
rs1499100	3	1177189	CNTN6
rs1499823	4	108258449	LEF1-AS1
rs151237	5	55236316	AC026704.1
rs151237	5	55236316	CCNO
rs151237	5	55236316	MCIDAS
rs1520148	3	151709165	-
rs153512	5	143589566	-
rs1540339	12	47863543	VDR
rs154100	5	152109163	LINC01933
rs1545300	1	111921382	KCND3
rs1548917	16	56075421	-
rs1559054	5	98111891	LINC01846
rs1560414	3	156723960	METTL15P1
rs1570193	14	88209316	KCNK10
rs157684	6	90488432	-
rs157982	10	78742740	-
rs1607237	3	179232509	KCNMB3
rs1607237	3	179232509	PIK3CA
rs1615942	12	12091288	BCL2L14
rs1622699	17	77288731	-
rs1622699	17	77288731	AC068594.1
rs1622699	17	77288731	SEPT9
rs163551	3	3084366	IL5RA
rs16836162	2	136708052	-
rs16840459	1	240646897	-
rs16840459	1	240646897	AL358176.3

rs16840459	1	240646897	AL358176.5
rs16840459	1	240646897	RNU5F-8P
rs16851872	3	141853201	-
rs16854065	2	167686410	-
rs16875467	5	5372744	ALG3P1
rs16899972	5	34998673	AGXT2
rs16936863	10	79113686	-
rs16936863	10	79113686	ZMIZ1
rs16969966	17	47241086	-
rs16988279	22	22425315	-
rs16988279	22	22425315	ASH2LP1
rs16988279	22	22425315	IGLV1-36
rs16988279	22	22425315	IGLV5-37
rs16988279	22	22425315	IGLVI-38
rs17031723	3	63400159	SYNPR
rs17102484	10	121712516	RPS15AP5
rs17115100	10	102831636	CYP17A1
rs17115100	10	102831636	CYP17A1-AS1
rs17115100	10	102831636	PFN1P11
rs17156818	7	82887678	PCLO
rs17185850	4	125007954	-
rs17232531	18	68612636	-
rs17288621	6	45935788	CLIC5
rs17326362	13	47323894	-
rs17359281	1	113643337	MAGI3
rs17501324	5	33853236	-
rs17501324	5	33853236	ADAMTS12
rs17590919	11	19166938	ZDHHC13
rs17656191	3	116563324	LINC00903
rs17656191	3	116563324	LSAMP
rs17663922	2	193342143	-
rs17692319	9	26538667	-
rs17694546	21	32648476	SYNJ1
rs17718041	5	147930126	EEF1GP2
rs17761563	14	56127069	AL138995.1
rs17761563	14	56127069	PELI2
rs17803363	9	18598638	ADAMTSL1
rs1783363	21	24127672	-
rs181050	7	88491600	-
rs1832741	9	107479248	KLF4
rs1832741	9	107479248	RNU6-492P
rs1834789	2	159081222	TANC1
rs1836698	15	99919229	-
rs1860461	7	6013633	AIMP2
rs1860461	7	6013633	EIF2AK1
rs1860461	7	6013633	PMS2
rs1860461	7	6013633	SNORA80D
rs1860461	7	6013633	Y_RNA
rs1867005	12	70584444	PTPRB
rs187238	11	112164265	AP002884.4
rs187238	11	112164265	AP002884.5
rs187238	11	112164265	IL18
rs187238	11	112164265	TEX12
rs1875112	3	113228648	BOC
rs1885322	6	38199380	BTBD9

rs1885322	6	38199380	SNORD45
rs1915480	8	12916449	-
rs1915613	10	64723459	-
rs1916011	7	150536953	ALDH7A1P3
rs1921984	2	216965941	AC007563.2
rs1939477	11	96195196	MAML2
rs1969578	4	159361939	RAPGEF2
rs198972	19	50876637	KLK2
rs198972	19	50876637	KLKP1
rs2013213	8	19721458	CSGALNACT1
rs2028088	3	86937633	-
rs2028088	3	86937633	VGLL3
rs2034860	17	79383558	RBFOX3
rs2051403	21	42752904	PDE9A
rs2052568	5	53118365	AC008966.1
rs2052568	5	53118365	MOCS2
rs2053984	14	49593304	-
rs2053984	14	49593304	AL139099.4
rs2053984	14	49593304	AL139099.5
rs2053984	14	49593304	LRR1
rs2053984	14	49593304	RHOQP1
rs2053984	14	49593304	RN7SL1
rs2053984	14	49593304	RPS29
rs2053984	14	49593304	Y_RNA
rs2070349	21	38053958	DSCR4
rs2073489	1	226875970	PSEN2
rs2073676	14	106510962	IGHV1-45
rs2073676	14	106510962	IGHV1-46
rs2073676	14	106510962	IGHV3-47
rs2073676	14	106510962	IGHVII-46-1
rs2073676	14	106510962	LINC00221
rs2073791	7	104482541	LHFPL3
rs2074783	7	29064261	CPVL
rs2074840	2	241202304	ANO7
rs2084274	10	69386138	HK1
rs2096735	11	96992442	MED28P5
rs2097983	19	57269727	-
rs2097983	19	57269727	AC005261.1
rs2097983	19	57269727	AC005261.4
rs2114600	2	125717854	LINC01889
rs212	7	27917875	-
rs212	7	27917875	JAZF1
rs2121881	6	127588832	C6orf58
rs2122835	8	127266068	-
rs213299	3	60201000	FHIT
rs2144818	14	95276434	CLMN
rs2149070	13	102418792	-
rs2161044	3	175332679	NAALADL2
rs2170552	4	110247136	HSBP1P2
rs2173115	8	20244495	LZTS1
rs2196662	10	79015511	ZMIZ1-AS1
rs2217836	2	133445587	NCKAP5
rs2238329	15	90485169	IQGAP1
rs2247938	10	7455883	AL139125.1
rs2249250	9	35711809	MIR6852

rs2249250	9	35711809	TLN1
rs2269173	21	42771021	LINC01668
rs2269173	21	42771021	PDE9A
rs2269322	14	102983436	CDC42BPB
rs2270221	7	31864413	PDE1C
rs2279988	9	1042166	DMRT2
rs2279988	9	1042166	LINC01230
rs2282810	5	150173136	CDX1
rs2284975	21	42767634	PDE9A
rs2288359	10	60142265	AL592430.2
rs2288359	10	60142265	ANK3
rs2290595	5	55307994	-
rs2290595	5	55307994	DHX29
rs2290595	5	55307994	SKIV2L2
rs2292161	4	187660171	AC097521.1
rs2292161	4	187660171	LINC02492
rs230519	4	102545592	NFKB1
rs2305345	17	76044212	SRP68
rs2305448	12	44611041	NELL2
rs2311843	15	27938208	OCA2
rs2356929	1	209434376	-
rs2356929	1	209434376	MIR205
rs2356929	1	209434376	MIR205HG
rs2372946	2	217053159	-
rs2460438	6	114235907	HDAC2-AS2
rs2460438	6	114235907	HS3ST5
rs2494047	1	107670238	-
rs2494047	1	107670238	VAV3
rs2542197	2	216991469	-
rs2542197	2	216991469	AC007563.2
rs2542197	2	216991469	AC007749.1
rs2575763	3	87079022	-
rs2622777	15	51715426	AC020892.2
rs2622777	15	51715426	LYSMD2
rs2622777	15	51715426	SCG3
rs263547	9	17026931	AL162725.2
rs2647230	4	105271618	TET2
rs2704976	7	106424112	-
rs2704976	7	106424112	AC004917.1
rs2726459	4	105263440	TET2
rs2767322	1	117540966	VPS25P1
rs2791418	10	6918371	-
rs2791418	10	6918371	AL392086.1
rs281791	2	199985679	-
rs281791	2	199985679	MAIP1
rs2828176	21	23464190	-
rs2831982	21	28724071	-
rs2834079	21	33057864	AP000282.1
rs2834079	21	33057864	LINC00945
rs2834652	21	34857340	RUNX1
rs285483	1	165484976	AL157714.2
rs28658459	17	54878601	-
rs2869929	4	88322855	-
rs2869929	4	88322855	AC107067.1
rs2869929	4	88322855	Y_RNA

rs2898295	8	11738460	GATA4
rs2904649	8	17124934	AC079193.2
rs2904649	8	17124934	MICU3
rs29047	18	9989050	-
rs291695	20	33400396	CDK5RAP1
rs291695	20	33400396	SNTA1
rs2935644	5	2715920	LSINCT5
rs29475	7	111997185	DOCK4
rs2962271	5	51136344	-
rs2972469	3	115729274	GAP43
rs3010312	6	139438644	-
rs3012797	9	134932170	AL353611.1
rs3110232	5	38380913	EGFLAM
rs3110893	7	153513047	-
rs311734	17	6974080	AC027763.2
rs311734	17	6974080	ALOX12-AS1
rs315857	7	33880123	AC008080.3
rs3212219	5	159327453	AC008691.1
rs3212219	5	159327453	IL12B
rs3212219	5	159327453	RNU4ATAC2P
rs327518	7	127611738	FSCN3
rs327518	7	127611738	PAX4
rs330353	5	125600906	LINC02240
rs335305	4	61528042	-
rs335305	4	61528042	ADGRL3
rs341555	13	59796176	DIAPH3
rs34342262	2	175810557	-
rs35569752	12	115119411	-
rs369335	19	45341186	ERCC2
rs369335	19	45341186	KLC3
rs369335	19	45341186	RPS16P9
rs3737964	1	11806987	-
rs3737964	1	11806987	CLCN6
rs3737964	1	11806987	MTHFR
rs3743165	15	87861811	NTRK3
rs3744962	18	674320	ENOSF1
rs3744962	18	674320	TYMS
rs3751234	12	27782535	AC009511.2
rs3751234	12	27782535	KLHL42
rs3769381	2	168165805	-
rs3769381	2	168165805	STK39
rs3772078	2	215325122	ATIC
rs3780955	10	112407983	ACSL5
rs3780955	10	112407983	AL157786.1
rs3798105	5	133194937	AC010307.2
rs3798105	5	133194937	FSTL4
rs3828137	1	177951619	AL359075.2
rs3828137	1	177951619	SEC16B
rs3850167	2	176231580	-
rs3935338	16	81665482	CMIP
rs39837	5	56535038	AC022431.2
rs39837	5	56535038	C5orf67
rs4045957	8	269868	AC136777.2
rs4045957	8	269868	ZNF596
rs414099	9	17024443	AL162725.2

rs4145876	4	136081946	-
rs4146100	2	221200719	-
rs4148010	17	68876059	ABCA8
rs4242158	5	170577031	-
rs4242158	5	170577031	KCNIP1
rs4254222	14	48819836	AL512360.1
rs4316308	X	12700917	FRMPD4
rs432331	21	41368052	-
rs432331	21	41368052	MX2
rs4339079	3	72232762	LINC00877
rs4365324	17	10176840	GAS7
rs4370940	11	63417309	SLC22A9
rs4387324	11	99136384	CNTN5
rs4420311	12	27831257	uc_338
rs4474806	19	28728943	AC005394.2
rs4474806	19	28728943	AC005524.1
rs4483901	17	53784667	-
rs4518623	8	128247161	-
rs4522252	12	68355124	AC022511.1
rs4522252	12	68355124	LINC02384
rs4578435	12	113524480	-
rs4583435	2	10077825	AC104794.1
rs4583435	2	10077825	CYS1
rs4613538	4	47081703	GABRB1
rs4701015	5	181025560	-
rs4719709	7	22676905	-
rs4726572	7	142717016	PGBD4P1
rs4726572	7	142717016	TRBV27
rs4726572	7	142717016	TRBV28
rs4726572	7	142717016	TRBVB
rs4743683	9	104086191	SMC2
rs4743683	9	104086191	SMC2-AS1
rs4751111	10	129704731	AL157832.2
rs4751111	10	129704731	MGMT
rs4756345	11	36744675	-
rs4757144	11	13309679	ARNTL
rs4767751	12	118976105	SRRM4
rs4772237	13	99751198	CLYBL
rs4786125	16	6850546	RBFOX1
rs4793885	17	57650863	MSI2
rs4801848	19	50696816	SHANK1
rs480644	3	15083295	RBSN
rs4835128	4	148134565	NR3C2
rs4835488	4	148141042	AC069272.1
rs4835488	4	148141042	NR3C2
rs4838927	1	111920995	KCND3
rs4853036	2	69832692	ANXA4
rs4853036	2	69832692	GMCL1
rs4943130	13	33662579	AL139081.1
rs4943130	13	33662579	AL139383.1
rs494641	1	95555472	AL356479.1
rs4955720	3	170310812	-
rs4955720	3	170310812	PRKCI
rs4963463	11	62120535	INCENP
rs4978665	9	108002563	-

rs4979834	10	78712448	-
rs4980075	10	79403403	-
rs4980075	10	79403403	AL133481.1
rs4980075	10	79403403	ZCCHC24
rs4981042	12	103730283	STAB2
rs4984247	15	63466448	-
rs4984247	15	63466448	AC007950.1
rs5082	1	161223893	-
rs5082	1	161223893	APOA2
rs5082	1	161223893	FCER1G
rs5082	1	161223893	MIR5187
rs5082	1	161223893	NDUFS2
rs5082	1	161223893	NR1I3
rs5082	1	161223893	TOMM40L
rs508218	18	54253069	-
rs528715	6	23943901	-
rs562992	13	110256430	-
rs562992	13	110256430	AL161773.1
rs562992	13	110256430	COL4A1
rs5749450	22	32490490	FBXO7
rs5771716	22	48684963	FAM19A5
rs578268	10	8041925	AL390294.1
rs578268	10	8041925	GATA3-AS1
rs592242	10	78682714	-
rs592242	10	78682714	AC012560.1
rs5927492	X	30300689	NR0B1
rs5963418	X	38388267	AF241726.2
rs5963418	X	38388267	OTC
rs5963418	X	38388267	TDGF1P1
rs600671	15	79824890	AC021483.1
rs600671	15	79824890	MTHFS
rs600671	15	79824890	RNU6-667P
rs6012326	20	48051404	-
rs6014052	20	54558300	DOK5
rs6071089	20	36555296	DLGAP4-AS1
rs6071089	20	36555296	MYL9
rs6071089	20	36555296	Metazoa_SRP
rs613664	9	76771862	AL359314.1
rs613664	9	76771862	PCA3
rs613664	9	76771862	PRUNE2
rs629242	4	56276871	-
rs629242	4	56276871	KIAA1211
rs6464435	7	139891932	TBXAS1
rs6464953	7	149319034	AC004941.2
rs6465654	7	98156970	LMTK2
rs647997	11	132342158	NTM
rs6487927	12	30673401	IPO8
rs6498095	16	10606319	-
rs6504495	17	67283457	AC007448.1
rs6543146	2	102480236	SLC9A4
rs6551519	4	60118383	-
rs6564576	16	78690900	WWOX
rs6570423	6	140820820	-
rs6576443	15	25649511	-
rs6586793	8	19051997	PSD3

rs660207	13	102687015	METTL21C
rs660207	13	102687015	TPP2
rs6632367	X	35859135	AL590065.1
rs6667458	1	152174829	FLG-AS1
rs6667458	1	152174829	PUDPP2
rs6669481	1	205915146	SLC26A9
rs6690824	1	208522196	-
rs6692344	1	67530431	LINC01702
rs6740463	2	77556209	LRRTM4
rs6740584	2	207564627	CREB1
rs6747958	2	29834704	-
rs6747958	2	29834704	ALK
rs6758593	2	72485235	EXOC6B
rs6768773	3	197408948	-
rs6768773	3	197408948	BDH1
rs6780615	3	122876930	DIRC2
rs6787193	3	2394450	CNTN4
rs6855233	4	1008337	FGFRL1
rs6855233	4	1008337	IDUA
rs6865784	5	141837059	-
rs6896201	5	66506926	LINC02229
rs6897876	5	142308074	SPRY4
rs6897876	5	142308074	SPRY4-IT1
rs6897876	5	142308074	SPRY4-IT1_1
rs6897876	5	142308074	SPRY4-IT1_2
rs6900051	6	107844744	-
rs6900279	6	104611871	-
rs6923009	6	36327138	C6orf222
rs6930083	6	36666379	LAP3P2
rs6930083	6	36666379	PANDAR
rs6930083	6	36666379	Y_RNA
rs6938002	6	37558248	AL353597.1
rs6938002	6	37558248	AL353597.3
rs6938002	6	37558248	MIR4462
rs6958341	7	144869203	-
rs6962115	7	148615290	C7orf33
rs697212	12	103706839	STAB2
rs6973453	7	28320715	CREB5
rs7006985	8	9706927	TNKS
rs7014448	8	126978588	PCAT1
rs7023279	9	97760388	-
rs7040835	9	112580942	AL445187.1
rs7040835	9	112580942	KIAA1958
rs7044658	9	16519573	BNC2
rs7058587	X	29311579	IL1RAPL1
rs7109578	11	90135524	-
rs7109578	11	90135524	AP000648.2
rs7109578	11	90135524	NAALAD2
rs7114085	11	32217291	THEM7P
rs7117587	11	10798317	AC116535.2
rs7117587	11	10798317	EIF4G2
rs7117587	11	10798317	SNORD97
rs7118044	11	41500141	-
rs7147514	14	36955565	MIR4503
rs7147514	14	36955565	SLC25A21

rs7148287	14	20842987	-
rs7168443	15	92463819	AC090985.1
rs7168443	15	92463819	C15orf32
rs7168443	15	92463819	ST8SIA2
rs7168760	15	82898276	HOMER2
rs7212346	17	71563422	-
rs7212800	17	16975108	AC104024.1
rs7212800	17	16975108	TNFRSF13B
rs7237652	18	69843831	CD226
rs7237652	18	69843831	DOK6
rs730503	6	137817345	AL357060.2
rs7306174	12	93817689	CRADD
rs7317645	13	96478420	HS6ST3
rs7327741	13	85254510	-
rs7342054	10	56994887	-
rs7424968	2	62525786	AC092155.2
rs7459717	8	106499489	OXR1
rs7509377	20	2627355	-
rs7509377	20	2627355	TMC2
rs7554230	1	232621636	-
rs7556080	1	154666350	-
rs756944	17	69535094	MAP2K6
rs7602	1	65432268	LEPR
rs7602	1	65432268	LEPROT
rs7617307	3	177812122	LINC02015
rs761743	22	43365862	-
rs761878	22	49856029	AL117328.1
rs761878	22	49856029	AL117328.2
rs761878	22	49856029	Metazoa_SRP
rs761878	22	49856029	ZBED4
rs762039	14	20766692	EDDM3B
rs7628553	3	177807131	LINC02015
rs762880	22	33577429	LARGE1
rs763238	1	4683644	AJAP1
rs764514	8	71917923	MSC-AS1
rs7652402	3	77451697	ROBO2
rs7663401	4	105207797	TET2
rs7683416	4	105231827	TET2
rs769153	9	38436995	AL390726.6
rs7716934	5	170782091	AC008514.1
rs7716934	5	170782091	GABRP
rs7754188	6	160497382	LPAL2
rs7766597	6	154269298	IPCEF1
rs7785341	7	153470262	-
rs7791093	7	27923310	-
rs7791093	7	27923310	JAZF1
rs7794467	7	7647386	AC007161.3
rs7794467	7	7647386	RPA3
rs7794467	7	7647386	UMAD1
rs7797075	7	94485307	RNU6-1328P
rs7822888	8	70049194	PRDM14
rs787068	10	26519632	APBB1IP
rs787068	10	26519632	RNA5SP307
rs7906125	10	57596742	-
rs7983361	13	54278131	-

rs8005974	14	59145814	AL359219.1
rs8007176	14	21034186	AL161668.2
rs8007176	14	21034186	AL161668.3
rs8007176	14	21034186	NDRG2
rs8007176	14	21034186	RNASE13
rs8007176	14	21034186	RNASE7
rs8007176	14	21034186	TPPP2
rs8031107	15	81290527	IL16
rs8032397	15	91273542	SV2B
rs8041396	15	80364486	AC016705.2
rs8051403	16	84425042	ATP2C2
rs8098662	18	45184445	AC090376.1
rs8176725	9	133257230	ABO
rs8177656	10	5973021	IL15RA
rs8178071	8	47902027	PRKDC
rs8184921	21	21000405	NCAM2
rs827423	6	151835062	ESR1
rs834602	7	47406618	-
rs834602	7	47406618	TNS3
rs836896	19	20180454	AC011447.3
rs836896	19	20180454	BNIP3P16
rs836896	19	20180454	ZNF486
rs837492	12	124523485	-
rs837492	12	124523485	AC073592.1
rs837492	12	124523485	AC073592.7
rs837492	12	124523485	NCOR2
rs847907	7	12419003	-
rs848726	22	49346035	-
rs851994	6	151684409	ESR1
rs879850	9	88823329	-
rs886078	17	51658199	CA10
rs898549	10	43963266	-
rs905290	11	8732182	-
rs905290	11	8732182	ST5
rs910574	22	48695752	FAM19A5
rs913692	13	34546982	-
rs913692	13	34546982	AL161716.1
rs913692	13	34546982	LINC00457
rs913692	13	34546982	LINC02343
rs923799	10	103708137	SH3PXD2A
rs924273	1	61016272	NFIA
rs927012	6	106476583	-
rs927012	6	106476583	CRYBG1
rs927926	9	129246817	-
rs9282801	17	27769447	NOS2
rs9294937	6	168947678	-
rs9300005	11	29805667	AC107973.1
rs930016	15	48039040	AC092078.2
rs9309796	3	78260960	AC108752.1
rs9317872	13	70066513	KLHL1
rs9328514	6	951575	AL356130.2
rs9340770	6	151806967	AL356311.1
rs9340770	6	151806967	ESR1
rs9349437	6	47942202	PTCHD4
rs9365352	6	161919918	PRKN

rs9407751	9	16310725	-
rs940810	7	55209694	EGFR
rs9436303	1	65430991	-
rs9436303	1	65430991	LEPR
rs9436303	1	65430991	LEPROT
rs944725	17	27782545	NOS2
rs945134	6	116960016	-
rs9478170	6	151118562	AL138733.1
rs9479297	6	152337007	SYNE1
rs9525293	13	114181217	AL160396.1
rs9525293	13	114181217	AL160396.2
rs9530615	13	76966220	-
rs9530615	13	76966220	ACOD1
rs9543599	13	74363520	-
rs954705	8	23354711	-
rs954705	8	23354711	AC090197.1
rs954705	8	23354711	LOXL2
rs9566829	13	41715024	VWA8
rs9594670	13	42057353	DGKH
rs9598854	13	34256311	AL356321.1
rs9606990	22	32744668	SYN3
rs9610	11	118001371	IL10RA
rs9610	11	118001371	SMIM35
rs9610500	22	21866877	-
rs9610500	22	21866877	MAPK1
rs9651304	10	62912004	EGR2
rs9693239	8	102438913	-
rs978664	3	16741554	-
rs9806257	15	78103020	CIB2
rs9806257	15	78103020	SH2D7
rs9815301	3	24747528	AC092422.1
rs9840064	3	119280491	B4GALT4-AS1
rs9866684	3	24755033	AC092422.1
rs991115	9	14432099	-
rs997353	17	62148091	POLRMTP1

Table S6. Identified interacting SNPs from the merged KBCP and OBCS sample sets and their associated genes.

Variant name	Chromosome	Position start (bp)	Associated gene name
rs10238349	7	54684078	-
rs1048230	12	50992283	SLC11A2
rs10505505	8	127727525	CASC11
rs10505505	8	127727525	MYC
rs10745452	12	76239517	-
rs10908201	11	69726447	-
rs10962769	9	16959697	-
rs10963288	9	17815404	-
rs10982959	9	115857024	-
rs11002813	10	79097776	ZMIZ1
rs1107268	3	18158854	TBC1D5
rs11116572	12	84747209	-
rs11243124	6	6858918	-
rs11631945	15	50092016	ATP8B4
rs11707080	3	72092572	AC105265.1
rs11707080	3	72092572	AC105265.2
rs11707080	3	72092572	AC105265.4
rs11707080	3	72092572	LINC00877
rs11758492	6	115276953	-
rs11770876	7	101398827	AC004965.1
rs11770876	7	101398827	COL26A1
rs11858902	15	88324326	-
rs11880261	19	40282734	AC118344.1
rs11880261	19	40282734	AKT2
rs11880261	19	40282734	MIR641
rs11897836	2	43196776	-
rs12208557	6	12216887	-
rs12255398	10	121116364	-
rs12334945	8	89866677	-
rs12356887	10	125094601	CTBP2
rs12404261	1	91748278	TGFBR3
rs12441537	15	92015836	SLCO3A1
rs12479775	20	57469371	-
rs1261097	18	55201746	-
rs12746015	1	212463482	AC092803.1
rs12948848	17	13762771	COX10-AS1
rs12996863	2	232436106	DIS3L2P1
rs13230925	7	148188442	CNTNAP2
rs1324210	9	112028870	-
rs1324210	9	112028870	AL138756.1
rs1398883	17	43683024	AC004149.1
rs1402648	8	21000148	LINC02153
rs1436857	6	164071094	-
rs1485817	18	27068669	AQP4-AS1
rs1485817	18	27068669	CHST9
rs1487504	9	17000857	-
rs1543677	9	20854450	-
rs1543677	9	20854450	FOCAD
rs1548377	9	20631634	MLLT3
rs1594869	4	157541210	-
rs1609716	5	21758391	AC091946.1
rs1609716	5	21758391	CDH12

rs1649200	10	121484216	FGFR2
rs16905251	8	134782084	AC083843.2
rs16905251	8	134782084	AC083843.3
rs16969966	17	47241086	-
rs1704740	1	162752426	DDR2
rs17079819	8	3303217	CSMD1
rs17191656	16	6055650	AC009135.1
rs17191656	16	6055650	RBFOX1
rs17335631	2	43646366	PLEKHH2
rs17352281	1	145876257	ANKRD35
rs17373027	15	84136219	EFTUD1P1
rs17384175	2	201597483	C2CD6
rs17452586	4	15141865	AC098829.1
rs175207	22	20189538	AC007663.2
rs17649956	4	135640153	-
rs17695685	7	27959537	JAZF1
rs1868272	2	46090156	PRKCE
rs1990629	16	49683660	ZNF423
rs2038047	1	176916170	ASTN1
rs2055953	1	41759909	HIVEP3
rs2115299	19	48044939	CABP5
rs2115299	19	48044939	PLA2G4C
rs2228083	9	113390660	ALAD
rs225215	17	32569892	-
rs225215	17	32569892	MYO1D
rs2279525	4	23792629	PPARGC1A
rs237597	10	27340951	AL355493.1
rs237597	10	27340951	AL355493.2
rs237597	10	27340951	FAM210CP
rs237597	10	27340951	RNU6-452P
rs2391191	13	105467097	DAOA
rs2391191	13	105467097	DAOA-AS1
rs2391191	13	105467097	DAOA-AS1_1
rs2391191	13	105467097	DAOA-AS1_2
rs2414505	15	57389101	CGNL1
rs2420941	10	121470112	FGFR2
rs2438085	2	105264030	TGFBRAP1
rs2518172	6	101348083	-
rs263640	9	17019123	AL162725.2
rs284168	1	91747678	TGFBR3
rs2897058	5	86471226	-
rs2982560	6	151734471	ESR1
rs303169	10	89435606	SLC16A12
rs3861059	11	42429196	-
rs4054823	17	13721707	-
rs41098	5	141640533	FCHSD1
rs41098	5	141640533	HDAC3
rs41098	5	141640533	RELL2
rs414099	9	17024443	AL162725.2
rs4147101	10	110779004	RBM20
rs4393800	2	45106935	-
rs4479723	4	132163449	-
rs45631608	10	121598759	-
rs45631608	10	121598759	FGFR2
rs4599845	8	115581371	TRPS1

rs4646992	17	48567980	-
rs4646992	17	48567980	HOXB-AS2
rs4646992	17	48567980	HOXB-AS3
rs4646992	17	48567980	HOXB3
rs4646992	17	48567980	HOXB4
rs4696737	4	8042497	ABLIM2
rs4705028	5	147244014	STK32A
rs4705028	5	147244014	STK32A-AS1
rs473539	19	56085449	LINC01864
rs473539	19	56085449	ZNF787
rs4826962	X	107358915	DNAJA1P3
rs5369	6	12294025	EDN1
rs570336	13	33371212	AL139383.1
rs5950350	X	20326738	AL928596.1
rs6014870	20	56897243	-
rs6075521	20	19507073	SLC24A3
rs6437581	3	105344372	-
rs6501668	17	73869392	-
rs6561355	13	47140322	-
rs6598531	15	98586884	-
rs6673186	1	145867640	ANKRD35
rs6673186	1	145867640	PIAS3
rs6678420	1	91718696	TGFBR3
rs6757782	2	152421985	-
rs6757782	2	152421985	FMNL2
rs6777133	3	24160620	-
rs6777133	3	24160620	THRB
rs6870714	5	2784958	AC116359.1
rs6925243	6	34414160	RPS10
rs6925243	6	34414160	RPS10-NUDT3
rs6938002	6	37558248	AL353597.1
rs6938002	6	37558248	AL353597.3
rs6938002	6	37558248	MIR4462
rs7184855	16	6901258	RBFOX1
rs720437	7	27878796	JAZF1
rs721265	6	79783217	-
rs7214412	17	71103686	CASC17
rs7311263	12	943227	-
rs7311263	12	943227	RAD52
rs750439	1	156460957	AL139412.1
rs750439	1	156460957	MEF2D
rs759103	6	139447032	-
rs7687342	4	88848863	FAM13A
rs7744152	6	139342748	AL592429.1
rs7782228	7	16995193	-
rs7811660	7	10082818	-
rs7947571	11	132445834	-
rs7947571	11	132445834	OPCML
rs8042229	15	31776097	-
rs8042229	15	31776097	AC026951.1
rs8042229	15	31776097	OTUD7A
rs8068256	17	53624699	-
rs8176725	9	133257230	ABO
rs836959	12	49881354	FAIM2
rs851027	6	36023098	MAPK14

rs851027	6	36023098	SLC26A8
rs881686	4	5721418	EVC
rs9295077	6	168613331	SMOC2
rs9296461	6	45583734	RUNX2
rs9296461	6	45583734	RUNX2-AS1
rs931318	3	59924949	FHIT
rs9514371	13	105125866	-
rs9564803	13	71294227	RABEPKP1
rs957645	11	11096939	-
rs9855757	3	153150457	AC117394.2
rs9920270	15	78373946	AC011270.1
rs998204	12	127881506	AC087894.1
rs998204	12	127881506	LINC02393

Table S7. 51 known breast cancer associated SNPs and their associated genes.

Variant name	Chromosome	Position start (bp)	Associated gene name
rs1011970	9	22062135	CDKN2B-AS1
rs10472076	5	58888234	-
rs1053338	3	63982224	ATXN7
rs10759243	9	107543834	AL389915.1
rs10759243	9	107543834	PPIAP88
rs10771399	12	28002147	-
rs11075995	16	53821379	-
rs11075995	16	53821379	FTO
rs11199914	10	121334387	-
rs11242675	6	1318643	-
rs11242675	6	1318643	FOXQ1
rs11242675	6	1318643	LINC01394
rs11571833	13	32398489	BRCA2
rs11571833	13	32398489	N4BP2L1
rs11780156	8	128182395	-
rs11814448	10	22026914	-
rs11820646	11	129591276	AP003500.1
rs12422552	12	14260997	-
rs12422552	12	14260997	GNAI2P1
rs12662670	6	151597721	CCDC170
rs12710696	2	19121042	-
rs12710696	2	19121042	LINC01376
rs1292011	12	115398717	-
rs132390	22	29225488	-
rs132390	22	29225488	EMID1
rs13329835	16	80616908	CDYL2
rs1353747	5	59041654	AC092343.1
rs1353747	5	59041654	PDE4D
rs1432679	5	158817075	EBF1
rs1436904	18	26990703	AQP4-AS1
rs1436904	18	26990703	CHST9
rs1550623	2	173348166	AC092573.2
rs1550623	2	173348166	CDCA7
rs17356907	12	95633983	-
rs17356907	12	95633983	PGAM1P5
rs17529111	6	81418669	-
rs17817449	16	53779455	FTO
rs2046210	6	151627231	CCDC170
rs2363956	19	17283315	-
rs2363956	19	17283315	ABHD8
rs2363956	19	17283315	AC010463.1
rs2363956	19	17283315	ANKLE1
rs2363956	19	17283315	BABAM1
rs2363956	19	17283315	MRPL34
rs2363956	19	17283315	USHBP1
rs2588809	14	68193711	RAD51B
rs2736108	5	1297373	TERT
rs2823093	21	15148511	-
rs2981579	10	121577821	FGFR2
rs3803662	16	52552429	CASC16
rs3803662	16	52552429	TOX3
rs3817198	11	1887776	AC051649.2

rs3817198	11	1887776	LSP1
rs3817198	11	1887776	MIR7847
rs4245739	1	204549714	MDM4
rs4808801	19	18460331	-
rs4808801	19	18460331	ELL
rs4849887	2	120487546	-
rs527616	18	26757460	AQP4-AS1
rs554219	11	69516874	-
rs6001930	22	40480230	-
rs6001930	22	40480230	MKL1
rs616488	1	10506158	PEX14
rs6504950	17	54979110	STXBP4
rs6762644	3	4700592	ITPR1
rs704010	10	79081391	-
rs704010	10	79081391	ZMIZ1
rs7072776	10	21744013	MLLT10
rs7726159	5	1282204	TERT
rs7904519	10	113014168	TCF7L2
rs865686	9	108126198	-
rs889312	5	56736057	-
rs941764	14	91374725	-
rs941764	14	91374725	CCDC88C
rs9693444	8	29652100	-
rs999737	14	68567965	RAD51B

References

1. Chen, T. & Guestrin, C. XGBoost: A scalable tree boosting system. In *Proc. of KDD*, 785–794 (2016).
2. Buja, A., Stuetzle, W. & Shen, Y. Loss functions for binary class probability estimation and classification: Structure and applications. *Technical Report, University of Pennsylvania* (2005).
3. Breiman, L., Friedman, J., Stone, C. & Olshen, R. A. *Classification and regression trees* (Taylor & Francis, 1984).
4. Friedman, J. H. Greedy function approximation: a gradient boosting machine. *Annals of Statistics* **29**, 1189–1232 (2001).
5. Michailidou, K. *et al.* Genome-wide association analysis of more than 120,000 individuals identifies 15 new susceptibility loci for breast cancer. *Nature Genetics* **47**, 373–380 (2015).
